# BODIPY-directed dynamic covalent templating of carbon nanohoop rotaxanes with near-unity Förster resonance energy transfer

**DOI:** 10.1039/d6sc02763f

**Published:** 2026-07-28

**Authors:** Shengzhu Guo, Siwei Wu, Yan-Qing Fan, Le-Ping Zhang, Zhe Lian, Xiaonan Li, Ying Wang, Hua Jiang

**Affiliations:** a College of Chemistry, Beijing Normal University Beijing 100875 P. R. China jiangh@bnu.edu.cn; b Department of Chemistry, Xinjiang Normal University Urumqi 830054 China

## Abstract

Mechanically interlocked carbon nanostructures remain an underexplored frontier in carbon nanoscience, largely due to the substantial synthetic challenges inherent to constructing such topologically complex architectures. Herein, we report a BODIPY-directed dynamic covalent templating strategy for the efficient assembly of rotaxanes. In this approach, F-BODIPY is installed onto a carbon nanohoop bearing a 2,2′-biphenol unit *via* nucleophilic substitution, affording a pseudo-rotaxane O-BODIPY derivative that serves as a key intermediate. Subsequent Sonogashira coupling with acetylenic stoppers yields the pre-rotaxane RxOB-1. Hydrogenation of the alkyne units in RxOB-1 produces RxOB-2, during which the O-BODIPY moiety undergoes cleavage and reconstitution to regenerate the F-BODIPY unit, ultimately delivering the target rotaxane RxFB-2. The structures of O-BODIPY intermediate 6 and RxOB-1 were unambiguously established by single-crystal X-ray diffraction analysis. Photophysical studies reveal that these (pre)rotaxanes exhibit high fluorescence quantum yields of up to 0.78, along with highly efficient Förster resonance energy transfer (FRET), with calculated energy transfer efficiencies approaching unity. Collectively, this work establishes a versatile synthetic platform for accessing mechanically interlocked carbon nanostructures.

## Abstract 2

Cycloparaphenylenes/BODIPY-based rotaxanes were synthesized through detachable template strategy, exhibiting high quantum yields of up to 0.78 and highly efficient FRET, with calculated energy transfer efficiencies approaching 100%.

## Graphical abstract

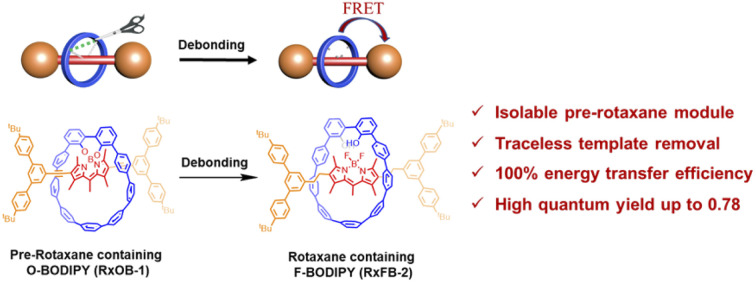

## Introduction

**Fig. 1:**
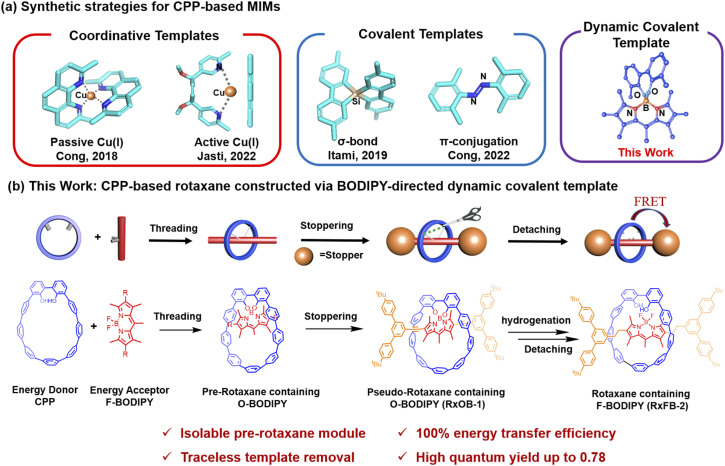
Synthetic strategies for CPP-based MIMs (a) and the CPP/BODIPY-based rotaxane in this work (b).

The distinctive size-dependent photophysical properties of cycloparaphenylenes ([*n*]CPPs), arising from their radially conjugated π-systems, render this class of macrocycles a uniquely versatile platform.^1–6^ Consequently, [*n*]CPPs have attracted considerable attention for applications spanning chiral recognition,^7^ hierarchical self-assembly,^8–11^ organic electronic devices,^12–21^ fluorescence imaging,^22,23^ circularly polarized luminescence,^24–35^ and beyond.^36–39^ With the continued expansion of synthetically tailored [*n*]CPP architectures, mechanically interlocked molecules (MIMs) incorporating [*n*]CPPs have emerged as particularly challenging yet highly rewarding targets at the forefront of contemporary research. To date, both covalent and coordinative templating strategies have been developed for the construction of [*n*]CPP-based MIMs (Fig. 1a).^40–48^ For example, the Cong group^40^ and the Jasti group^42^ employed copper(i) complexes as passive and active templates, respectively, to access CPP-based [2]catenanes. Meanwhile, the Itami group^41^ and the Cong group^43^ introduced spirosilane as a removable σ-covalent template and an azo unit as a traceless π-covalent template, respectively, enabling the synthesis of CPP [2]catenanes and trefoil. Beyond these seminal contributions, several elegant approaches toward analogous [2]catenanes and rotaxanes derived from topologically complex nanocarbons have also been reported.^44–54^ Despite these advances, progress in this area remains limited, underscoring the inherent challenges associated with their synthesis and purification, and highlighting the urgent need for innovative strategies to access CPP-derived MIMs.

Dynamic covalent bonds have been widely exploited in self-healing materials, adaptive and stimuli-responsive systems, error-correcting supramolecular assemblies, sensing, and reversible molecular recognition.^55–57^ Their unique ability to combine structural robustness with reversible bond exchange also makes them particularly attractive as covalent templates for the construction of mechanically interlocked molecules (MIMs), enabling efficient assembly followed by bond cleavage under mild and orthogonal conditions.^55^ Among the various dynamic covalent motifs, boron-based linkages—including boronic esters as well as reversible B–N and B–O coordination bonds—are especially appealing because their reversible condensation and coordination chemistry provides versatile and highly controllable platforms for dynamic self-assembly.^58^ Despite these attractive characteristics, boron chemistry remains surprisingly underutilized in the synthesis of MIMs. Although a number of boron-containing interlocked molecules have been reported in which boron functionalities are introduced only after formation of the mechanical bond,^59–63^ the deliberate use of boron as a structural component or dynamic templating motif to direct the assembly of interlocked architectures has received remarkably limited attention.^64–66^ This discrepancy highlights an important opportunity to exploit the unique reversibility and directional coordination chemistry of boron for the construction of mechanically interlocked systems.

Recent studies have begun to demonstrate the considerable potential of boron-directed assembly strategies. Schaufelberger and co-workers pioneered the use of dynamic boronic ester templation for rotaxane synthesis, while simultaneously exploiting the boron center as a versatile synthetic handle for post-assembly functionalization.^64^ In parallel, Trolez and co-workers demonstrated that tetracoordinate boron centers can efficiently mediate the threading of BODIPY and boron β-diketonate chromophores through macrocyclic hosts, leading to the first examples of BODIPY-based pseudorotaxanes and boron β-diketonate-derived rotaxanes.^65,66^ These pioneering studies collectively establish the feasibility of boron-directed threading while revealing the largely untapped potential of boron-containing chromophores as dynamic covalent templates for mechanically interlocked architectures. Among these systems, BODIPY is particularly attractive as a next-generation templating platform. Beyond its outstanding photophysical properties, the reversible B–N coordination chemistry, well-defined tetrahedral boron center, and exceptional structural modularity provide a unique combination of dynamic reversibility and geometric precision. These characteristics make BODIPY an ideal scaffold for developing new dynamic covalent templating strategies and expanding the structural diversity and functional complexity of mechanically interlocked molecules.

Inspired by the viability of boron-directed threading strategies as well as versatile chemistry underlying F-/O-BODIPY interconversion,^67–71^ we herein present a BODIPY-directed dynamic covalent templating strategy for the construction of CPP-based rotaxanes. In this approach, F-BODIPY serves as a dynamic covalent template that coordinates with a conjugated carbon nanohoop containing a 2,2′-biphenol unit through substitution of the fluorine atoms, thereby generating an O-BODIPY-containing pseudorotaxane that can be isolated as a key intermediate (Fig. 1b). Subsequent stoppering transforms this intermediate into a pre-rotaxane. Upon further treatment, the O-BODIPY moiety is cleaved from the carbon nanohoop through disruption of the B–N coordination bonds, releasing a dipyrrin-based axle. The latter can then be readily reconverted into the F-BODIPY form upon treatment with triethylamine and BF_3_·Et_2_O, ultimately affording the target rotaxane incorporating an F-BODIPY unit. The resulting (pre)rotaxanes exhibit pronounced fluorescence properties, with quantum yields of up to 0.78. Moreover, efficient intramolecular Förster resonance energy transfer (FRET) is observed, with energy-transfer efficiencies approaching unity.

## Results and discussion

**Scheme 1:**
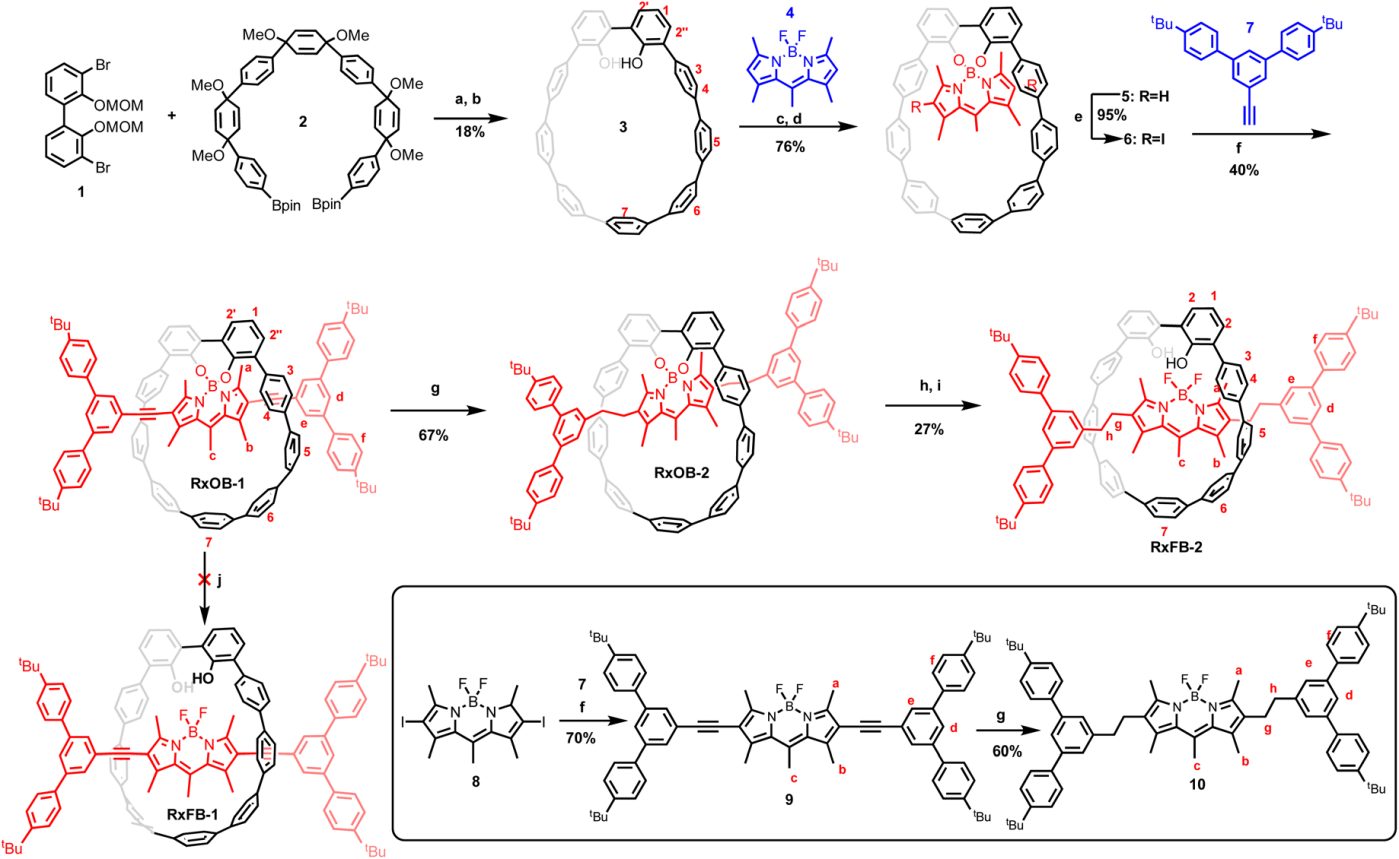
Synthetic routes of rotaxane. (a) 2 M K_2_CO_3_, Pd(PPh_3_)_4_, dioxane, 115 °C, overnight; (b) H_2_SnCl_4_/THF, RT, overnight; (c) AlCl_3_, DCM, RT, 1 h; (d) toluene, 115 °C, 2 days; (e) DCM, NIS, RT, overnight; (f) Pd(PPh_3_)_2_Cl_2_, CuI, Et_3_N, THF, RT, overnight; (g) Pd/C, THF/EA, 50 °C, 1.5 MPa, overnight; (h) DCM, CH_3_SO_3_H, −30 °C, 2h; (i) Et_3_N, BF_3_·OEt_2_, , overnight; (j) see SI.

As depicted in Scheme 1, methoxymethyl (MOM)-protected 3,3′-dibromo-2,2′-biphenol (1) underwent Suzuki coupling with C-shaped precursor 2 in the presence of Pd(PPh_3_)_4_ and aqueous K_2_CO_3_ (2 M), followed by reductive aromatization to afford nanohoop 3 bearing two pendant hydroxyl groups for boron coordination, in 18% yield over two steps. Subsequently, BODIPY 4 was treated with AlCl_3_ in anhydrous dichloromethane under reflux for 2 h to generate the corresponding activated intermediate, which, upon addition of a solution of 3 in toluene and prolonged reflux for 2 days, furnished compound 5 in 75% yield. Iodination of 5 with NIS proceeded smoothly to give compound 6 in 95% yield. Sonogashira coupling of 6 with 7 then delivered the pre-rotaxane RxOB-1 in 40% yield.

Attempts to directly convert the O-BODIPY unit in RxOB-1 into the corresponding F-BODIPY motif to afford rotaxane RxFB-1 were unsuccessful. No debonding of the O-BODIPY moiety was observed under either basic conditions or photolysis (see SI, Table S1), and RxOB-1 was quantitatively recovered, indicating its notable chemical robustness. Further attempts involving treatment with methanesulfonic acid, followed by triethylamine and BF_3_·Et_2_O, also failed to yield the desired product; instead, decomposition of RxOB-1 was observed, likely due to the instability of the alkyne moieties under acidic conditions. To address this limitation, the alkyne units in RxOB-1 were hydrogenated using Pd/C, affording the saturated pre-rotaxane RxOB-2 in 67% yield. Gratifyingly, the O-BODIPY unit in RxOB-2 could be efficiently cleaved under acidic conditions to release the dipyrrin-based axle. Subsequent *in situ* treatment with triethylamine and BF_3_·Et_2_O enabled reformation of the F- BODIPY motif, thereby furnishing the target rotaxane RxFB-2. This successful interconversion of O-BODIPY to F-BODIPY highlights the potential of this strategy for the construction of structurally diverse rotaxane architectures. In parallel, axle model compounds 9 and 10 were prepared to facilitate detailed photophysical investigations of the individual ring and axle components, as well as the resulting interlocked systems.

**Fig. 2:**
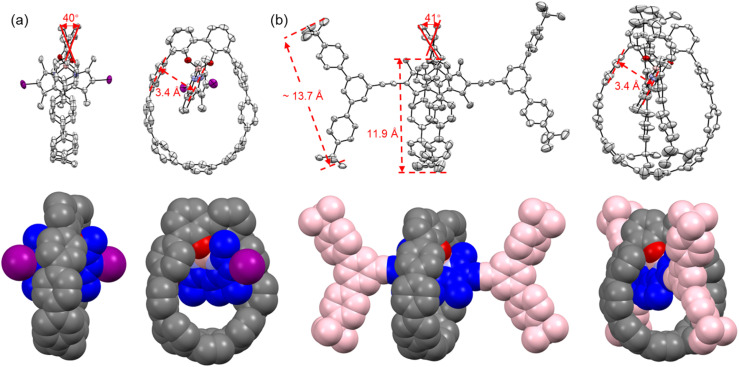
X-Ray structure (ellipsoidal representation in 50% probability) and spacefill representation of compound 6 (a) and RxOB-1 (b) in face view and lateral view; solvent molecules and hydrogens were removed for clarity.

The structures of compound 6 and RxOB-1 were further elucidated by single-crystal X-ray diffraction analysis (Fig. 2).^72^ Suitable single crystals of both compounds were obtained by slow diffusion of ethanol into their respective dichloromethane or chloroform solutions. The crystallographic data unambiguously confirm the expected connectivity as well as the mechanically interlocked architecture in RxOB-1. Both compound 6 and RxOB-1 crystallize in the monoclinic *P*1̄ space group, with two pairs of enantiomers present in each unit cell. Structural analysis clearly reveals that the BODIPY unit is anchored within the macrocyclic cavity through two B–O bonds. Notably, the BODIPY core adopts a tilted orientation, which enables additional stabilization *via* π–π interactions with a benzene ring of the macrocycle, as evidenced by a centroid-to-centroid distance of 3.4 Å. Furthermore, the distance between the two *tert*-butyl groups on the same stopper in RxOB-1 (*ca.* 13.7 Å) is sufficiently large to effectively prevent slippage of the carbon nanohoop. This structural feature is expected to ensure retention of the mechanical bond following removal of the BODIPY template.

**Fig. 3:**
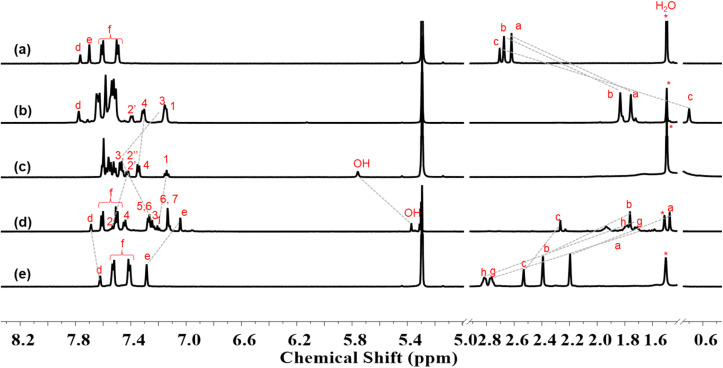
Partial ^1^H NMR spectra (2 M in CD_2_Cl_2_, 600 MHz, 293 K) of compound (a) 9, (b) RxOB-1, (c) carbon nanohoop 3, (d) RxFB-2 and (e) 10. The asterisks indicate water peaks.

With the target molecules in hand, comparative ^1^H NMR analyses were performed (Fig. 3). Notably, the proton signals of RxOB-1 exhibit pronounced upfield shifts relative to those of axle model compound 9, particularly for the BODIPY-associated protons (H_a_, H_b_, and H_c_). Specifically, H_a_, H_b_, and H_c_ in RxOB-1 (Fig. 3b) are shifted upfield by Δ*δ* = −0.86, −0.84, and −2.01 ppm, respectively, compared to those in 9 (Fig. 3a). These substantial shielding effects are indicative of strong through–space interactions between the axle and macrocyclic components, consistent with the formation of an interlocked, conjugated architecture. A similar trend is observed for RxFB-2 (Fig. 3d) in comparison with axle model 10 (Fig. 3e). The BODIPY protons (H_a_, H_b_, and H_c_) in RxFB-2 display upfield shifts of Δ*δ* = −0.72, −0.63, and −0.26 ppm, respectively. In addition, the methylene protons (–CH_2_–) linking the BODIPY core and the benzene ring exhibit an upfield shift of approximately Δ*δ* = −1.0 ppm. The hydroxyl proton in RxFB-2 appears at 5.37 ppm, corresponding to an upfield shift of Δ*δ* = −0.39 ppm relative to nanohoop 3 (Fig. 3c), further supporting successful debonding of the O-BODIPY unit. Importantly, ^19^F NMR analysis (Fig. S4a) of RxFB-2 confirms the reformation of the F-BODIPY motif. Moreover, the fluorine resonance displays a downfield shift of up to Δ*δ* = 0.59 ppm relative to that of axle 10 (Fig. S4b), further supporting the successful formation of the target rotaxane. Comparable chemical shift trends are also observed for compounds 5, 6, and RxOB-2, as well as their corresponding axle and macrocycle components (Fig. S1–S3), further supporting the generality of these observations.

**Fig. 4:**
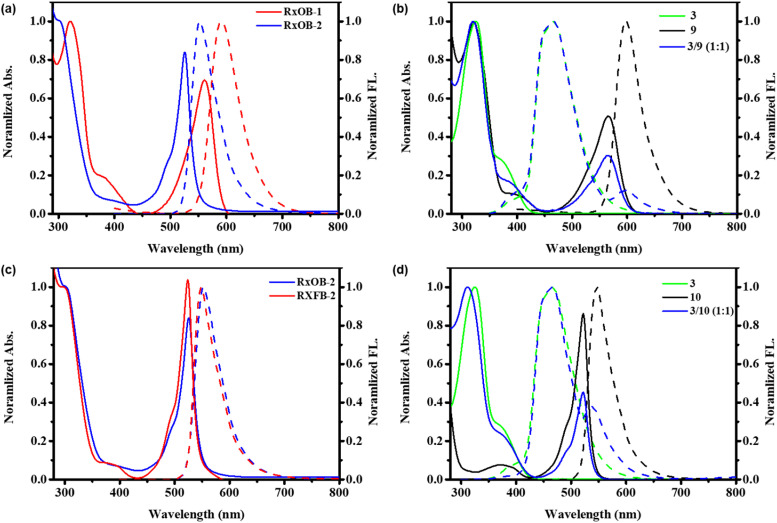
UV/Vis absorbance (solid line) and fluorescence emission (dashed line) spectra of (a) RxOB-1 and RxOB-2, (b) compounds 3, 9, and the equimolar mixture of compounds 3 and 9, (c) RxOB-2 and RxFB-2, (d) compounds 3, 10 and the equimolar mixture of compounds 3 and 10, in CH_2_Cl_2_ (10^−5^ M, *λ*_ex_ = 320 nm).

The photophysical properties of the (pre)rotaxanes and their individual components (wheel and axle) were systematically investigated, and the corresponding data are summarized in Fig. 4 and Table 1. The UV-vis absorption spectrum of RxOB-1 exhibits two prominent bands at 324 and 564 nm (Fig. 4a). The isolated wheel 3 displays an intense absorption at 325 nm along with a broad shoulder spanning ∼365–420 nm, whereas axle 9 shows characteristic absorptions at 320 and 568 nm (Fig. 4b). Notably, the absorption profile of RxOB-1 closely matches that of an equimolar mixture of 3 and 9 (Fig. 4c), suggesting the absence of ground-state charge transfer interactions. In contrast, RxOB-2 and RxFB-2 (Fig. 4d) display nearly identical absorption features, with two bands centered at 300 and 523 nm. These bands are blue-shifted relative to those of RxOB-1 (Fig. 4a), which can be attributed to a reduced degree of conjugation following hydrogenation. Importantly, the enhanced absorption intensity at 523 nm observed for RxFB-2, compared to RxOB-2, further supports the formation of the rotaxane structure.

**Table 1:** Summary of the photophysical characteristics of the investigated compounds

Compd	3	RxOB-1	RxOB-2	RxFB-2	9	10
*λ*_abs_ (nm)	325	324, 564	300, 523	300, 523	320, 568	522
*ε*_m_ (×10^4^ M^−1^·cm^−1^)	8.02	8.02, 5.61	8.03, 6.73	8.03, 8.35	7.13	7.55
*λ*_em_ (nm)	476	594	551	551	598	548
*Φ*_F_	0.86	0.78	0.42	0.41	0.63	0.47
*τ*(ns)	2.60	2.8	6.69	6.54	2.23	7.36
*k*_r_ (×10^8^ s^−1^)	3.31	3.75	0.63	0.87	2.83	0.64
*k*_nr_ (×10^8^ s^−1^)	0.54	1.06	0.63	0.90	1.66	0.72
*Φ*_ET_	—	100%	100%	100%	—	—

This enhancement is consistent with the higher molar extinction coefficient of the F-BODIPY unit in RxFB-2 relative to the O-BODIPY counterpart in RxOB-2 (Table 1).^73^

In the fluorescence spectra, wheel 3 and axle 9 each display a single emission band at 467 nm (*Φ* = 0.86, *τ* = 2.60 ns) and 598 nm (*Φ* = 0.63, *τ* = 2.23 ns), respectively, upon excitation at 320 nm (Fig. 4b). In contrast, an equimolar mixture of 3 and 9 exhibits a dominant emission at 467 nm with only a very weak band at 598 nm under identical conditions (Fig. 4c), indicating the absence of intermolecular Förster resonance energy transfer (FRET). Strikingly, the pre-rotaxane RxOB-1 displays a single emission band at 592 nm (*Φ* = 0.78, *τ* = 2.08 ns) upon excitation at 320 nm (Fig. 4a), which can be at-tributed to emission from the BODIPY axle. The complete quenching of the wheel emission, together with the substantial spectral overlap between the emission of 3 and the absorption of 9, indicates highly efficient intramolecular FRET within RxOB-1 (*Φ*_ET_ ≈ 100%, Table 1), with the wheel and axle serving as donor and acceptor, respectively. The near-unity energy transfer efficiencies appear to result from the rotaxane structure,^41^ which confines the energy donor and acceptor in close proximity.

Similarly, RxOB-2 also exhibits efficient intramolecular FRET, giving rise to a single emission band at 550 nm that is blue-shifted relative to RxOB-1. Notably, the rotaxane RxFB-2 likewise displays a single emission centered at 550 nm, closely resembling that of RxOB-2.

These observations not only confirm the persistence of highly efficient intramolecular FRET in RxFB-2, but also provide further evidence for the successful formation of the mechanically interlocked architecture.^44^ The fluorescence quantum yields of RxOB-2 and RxFB-2 were determined to be 0.42 and 0.41, with corresponding lifetimes of 6.69 and 6.54 ns, respectively. Furthermore, the radiative (*k*_r_) and nonradiative (*k*_nr_) decay rate constants for compounds 3, RxOB-1, RxOB-2, RxFB-2, and model systems 9 and 10 were calculated and are summarized in Table 1. Compounds 3, RxOB-1, and 9 exhibit *k*_r_ values that significantly exceed their *k*_nr_ counterparts, consistent with their high fluorescence quantum yields. In contrast, RxOB-2, RxFB-2, and 10 display *k*_r_ and *k*_nr_ values of comparable magnitude, resulting in moderately high emission efficiencies.

## Conclusions

In summary, we have developed a modular and versatile strategy for the construction of carbon nanohoop-based rotaxanes through the use of BODIPY as a dynamic covalent template. This approach enables efficient and controlled mechanical bond formation while providing access to structurally well-defined interlocked architectures. The resulting (pre)rotaxanes display excellent photophysical properties, including fluorescence quantum yields of up to 0.78 and highly efficient intramolecular Förster resonance energy transfer (FRET), with energy-transfer efficiencies approaching unity. Beyond enriching the structural diversity and supramolecular topology of mechanically interlocked molecules, this work highlights the unique potential of reversible BODIPY coordination chemistry as a powerful platform for templated synthesis. More broadly, the present strategy establishes a new conceptual framework for the programmable assembly of functional interlocked nanocarbon systems and is expected to facilitate the development of increasingly sophisticated mechanically interlocked architectures with tunable optoelectronic properties and advanced functions.

## Author contributions

S. G. synthesized and characterized all materials, and drafted the manuscript. S. W., L. Z., Z. L. and X. L. assisted with partial synthesis. Y. F. carried out partial 2D NMR. Y. W. reviewed and refined the manuscript. H. J. supervised the project, revised the manuscript, and acquired funding. All authors have given approval to the final version of the manuscript.

## Conflicts of interest

There are no conflicts to declare.

## Supplementary Material

SC-OLF-D6SC02763F-s001

SC-OLF-D6SC02763F-s002

## Data Availability

CCDC 2516995 and 2516996 contain the supplementary crystallographic data for this paper.^74*a*,*b*^ The data that supports the findings of this study are available in the supplementary information (SI) of this article. Supplementary information is available. See DOI: https://doi.org/10.1039/d6sc02763f.
