# Supplementary material for: BODIPY-directed dynamic covalent templating of carbon nanohoop rotaxanes with near-unity Förster resonance energy transfer
Source: Chem Sci. 2026 Jul 28. Online ahead of print. doi: 10.1039/d6sc02763f (PMC13410999; doi:10.1039/d6sc02763f)
Supplement: SC-OLF-D6SC02763F-s001 [file SC-OLF-D6SC02763F-s001.pdf]

Supplementary Information (SI) for Chemical Science.
This journal is © The Royal Society of Chemistry 2026

# BODIPY-Directed Dynamic Covalent Templating of Carbon Nanohoop Rotaxanes with Near-Unity Förster Resonance Energy Transfer

Shengzhu Guo,[a] Siwei Wu,[a] Yan-Qing Fan,[b] Le-Ping Zhang,[a] Zhe Lian,[a] Xiaonan Li,[a] Ying Wang,[a] Hua Jiang*[a]

[a] College of Chemistry, Beijing Normal University, Beijing 100875 (P. R. China), E-mail: jiangh@bnu.edu.cn.

[b] Department of Chemistry, Xinjiang Normal University, Urumqi 830054 (P. R. China).

**Table of Contents**

1. General Procedures and Materials ........................................................................2
2. Synthesis and characterization ............................................................................2
3. X-ray Crystallography ...........................................................................................7
4. Photophysical Properties ......................................................................................8
5. $^{1}H$, $^{13}C$ NMR and Ms Spectra.......................................................................11

## 1. General Procedures and Materials

**Materials and molecular characterization.** Anhydrous tetrafuran, dioxane, dichloromethane and toluene were gained from PureSolv MD 5 (Inert solvent purification system). Compound **1, 2, 4, 7, 8** were prepared according to previous work. Column chromatography was carried out on flash grade silica gel, using 1 - 20 psig pressure. Analytical TLC was carried out using tapered silica plates with a preadsorbent zone. NMR spectra were obtained with JEOL Delta (400 MHz and 600 MHz) using chloroform-d ($CDCl_3$) as solvents. The chemical shift references were as follows: ($^1H$) chloroform-*d*, 7.26 ppm; ($^{13}C$) chloroform-*d*, 77.00 ppm (chloroform-d). Mass spectra (ESI, MALDI) were acquired on GCT and FT-ICR spectrometer (Bruker Daltonics Inc. APEXII, BIFLEX III), respectively. UV-vis spectra were recorded on UV-2450 spectrophotometer. Fluorescence spectra were measured were obtained using and FS5 fluorescence spectrophotometer. The lifetime was measured by Edinburg FLS-980 fluorescence spectrometer.

## 2. Synthesis and characterization

**Synthesis of Compound 3:** To a degassed solution of C-shaped molecular scaffold **2** (418 mg, 0.46 mmol, 1.0 equiv.), **1** (200 mg, 0.46 mmol, 1.0 equiv.), and $K_2CO_3$ (634 mg, 4.6 mmol, 10 equiv.) in 260 mL of 25:1 THF/$H_2O$ was added $Pd(PPh_3)_4$ (46 mg, 0.046 mmol, 10 mol%) under nitrogen, then the mixture was degassed for 15 minutes. Thereafter, the mixture was heated at 75 °C for 48 hours under nitrogen atmosphere. The mixture was extracted with EA and the combined extracts were dried over anhydrous sodium sulfate. The crude product was used for the next step. A $H_2SnCl_4$/THF solution was freshly prepared by dissolving anhydrous $SnCl_2$▪$H_2O$ (496 mg, 2.2 mmol) in anhydrous THF (11 mL) under Ar atmosphere and then adding concentrated HCl (0.6 mL) to the solution. The resulting solution was deoxygenated and stirred for 0.5h. The crude product was dissolved in minimal anhydrous THF under a Ar atmosphere and the freshly prepared $H_2SnCl_4$/THF solution was added to this solution. The reaction mixture was stirred at room temperature for 24h before being quenched with NaOH/$H_2O$ solution. After concentrated under reduced pressure, the crude product was purified by flash column chromatography on silica gel eluting with DCM/PE (1/2) to afford compound as yellow powder (60 mg, 18% for two steps).

$^1H$ NMR (600 MHz, $CDCl_{3,}$ 298 K, ppm) δ 7.63 (s, 4H), 7.61-7.59 (m, 8H), 7.54-7.51 (m, 8H), 7.49-7.45 (m, 8H), 7.37-7.36 (d, J=6.0 Hz, 4H), 7.18-7.16 (t, 2H), 5.80 (s, 2H). $^{13}C$ NMR NMR (100 MHz, $CDCl_{3,}$ 298 K, ppm) δ149.3, 139.2, 139.1, 138.1, 138.0, 137.7, 137.5, 135.5, 131.1, 130.4, 129.9, 128.5, 127.9, 127.7, 127.7, 127.2, 126.8, 126.0, 121.8. HRMS (MALDI-TOF) calculated for $C_{54}H_{36}O_2$ $[M+H]^+$: 717.2710, found 717.2778.

**Synthesis of Compound 5:** A mixture of **4** (12 mg, 0.043 mmol) and aluminum chloride (15 mg, 0.108 mmol) in dry $CH_2Cl_2$ (2 mL) was stirred for 30 min at 40 °C under nitrogen atmosphere. The resulting solution was concentrated to ca. 0.2 mL under reduced pressure. The material containing was immediately used for the next step without further purification. The residue was redissolved in PhMe (5 mL) and **3** (28 mg, 0.039 mmol) was added. The mixture was heated at 120 °C for 2d. The solution was extracted with EA and washed with water and brine, and the organic layer was dried with anhydrous sodium sulfate. After concentrated under reduced pressure, the crude product was purified by flash column chromatography on silica gel eluting with DCM/PE (1/2) to afford compound as yellow powder (28 mg, 76%).

$^1H$ NMR (600 MHz, $CDCl_{3,}$ 298 K, ppm) δ 7.61-7.57 (m, 12H), 7.54-7.51 (m 10H), 7.37-7.36 (d, 2H), 7.27-7.26 (m, 4H), 7.16-7.14 (d, 4H), 7.11-7.10 (t, 2H), 5.47 (s,2H), 1.63 (s, 6H), 1.61 (s, 6H), 0.52 (s,3H). $^{13}C$ NMR NMR

(100 MHz, $CDCl_3$, 298 K, ppm) δ 153.3, 152.6, 138.6, 138.3, 138.1, 138.0, 137.8, 137.4, 136.4, 134.5, 132.2, 131.3, 130.5, 129.9, 129.1, 128.8, 128.2, 127.9, 127.8, 127.4, 127.3, 127.1, 126.9, 125.0, 121.2, 120.9, 16.9, 15.3. HRMS (MALDI-TOF) calculated for $C_{68}H_{51}BN_2O_2$ $[M+H]^+$: 939.4127, found 939.4128.

**Synthesis of Compound 6:** To a solution of **5** (28 mg, 0.003 mmol) in 3 mL of anhydrous dichloromethane was added N-iodosuccinimide (17 mg, 0.075 mmol) at room temperature under nitrogen atmosphere overnight. The mixture was extracted with DCM and the combined extracts were dried over anhydrous sodium sulfate. After concentrated under reduced pressure, the crude product was purified by flash column chromatography on silica gel eluting with PE/DCM=2:1 to afford compound as red solid (34 mg, 95%).

1H NMR (400 MHz, $CDCl_3$, 298 K, ppm) δ 7.59-7.47 (m, 26H), 7.40-7.38 (d, *J* = 7.6 Hz, 2H), 7.18-7.11 (m, 6H), 1.73 (s, 6H), 1.53 (s, 6H), 0.72 (s, 3H). $^{13}C$ NMR NMR (150 MHz, $CDCl_3$, 298 K, ppm) δ 153.8, 153.0, 141.0, 140.1, 138.4, 138.1, 138.0, 137.5, 136.5, 134.7, 131.9, 131.4, 130.5, 130.0, 128.4, 127.8, 127.7, 127.3, 127.3, 127.2, 124.9, 122.0, 27.0, 19.3, 17.0. HRMS (MALDI-TOF) calculated for $C_{68}H_{49}BI_2N_2O_2$ $[M+H]^+$: 1189.1915, found 1189.1978.

**Synthesis of RxOB-1:** To a 25 mL round bottom flask filled with nitrogen was added **6** (28 mg, 0.024 mmol), **7** (26 mg, 0.072 mmol), $Pd(PPh_3)_2Cl_2$ (2 mg, 0.0024 mmol), CuI (0.5 mg, 0.0024 mmol), dry THF (4 mL) and $Et_3N$ (2 mL) under nitrogen. The reaction mixture was stirred at room temperature for 24 h. The reaction mixture was then poured into a saturated $NaHCO_3$ solution and extracted with dichloromethane. The organic layers were combined and dried over anhydrous sodium sulfate. The organic solvent was removed under reduced pressure, and the residue was purified by column chromatography (PE/DCM=2/1) on silica gel to afford compound as red solid (16 mg, 40%)

$^1H$ NMR (400 MHz, $CDCl_3$, 298 K, ppm) δ 7.76 (s, 2H), 7.64-7.59 (m, 24H), 7.56-7.53 (m, 18), 7.42-7.40 (d, *J* = 7.6 Hz, 2H), 7.33-7.31 (d, *J* = 7.6 Hz, 4H), 7.17-7.14 (m, 6H), 1.84 (s 6H), 1.82 (s, 6H), 1.40 (s, 36H), 0.69 (s, 3H). $^{13}C$ NMR (125 MHz, $CDCl_3$, 298 K, ppm) δ 156.1, 152.8, 150.9, 142.0, 141.9, 141.8, 140.3, 138.4, 138.3, 138.0, 137.8, 137.7, 137.3, 136.4, 134.5, 132.0, 131.2, 130.5, 129.9, 128.4, 127.8, 127.7, 127.3, 127.2, 127.1, 126.9, 125.9, 125.0, 124.5, 121.8, 95.9, 83.0, 34.7, 31.4, 31.2, 29.7, 15.6, 15.3, 14.3. HRMS (MALDI-TOF) calculated for $C_{124}H_{107}BN_2O_2$ $[M+H]^+$: 1667.8464, found 1667.8508.

**Synthesis of RxOB-2: RxOB-1** (30 mg, 0.018 mmol) and Pd/C (10 mg, 10 wt % Pd) were loaded into a pressure reactor and EA (10 mL) and THF (5 mL) and a drop of acetic acid were added. The reaction mixture was stirred under pressure of hydrogen (15 atm) at 40 °C overnight, filtered and evaporated to give crude product. The crude product was purified by flash column chromatography on silica gel eluting with DCM/PE (1/2) to afford compound as red powder (20 mg, 67%).

$^1H$ NMR (600 MHz, $CDCl_3$, 298 K, ppm) δ 7.69 (s, 2H), 7.65-7.60 (m, 12H), 7.53-7.51 (d, *J* = 12.0 Hz, 8H ), 7.45-7.39 (m, 18H), 7.32-7.31 (d, *J* = 12.0 Hz, 4H ), 7.27-7.25 (d, *J* = 12.0 Hz, 2H), 7.18-7.16 (t, 2H), 7.08-7.07 (d, *J* = 5.8 Hz, 4H ), 7.02-7.00 (d, *J* = 11.8 Hz, 4H ), 2.72-2.69 (m, 4H), 2.56-2.53 (m, 4H), 1.66 (s, 3H), 1.57 (s, 3H), 1.38 (s, 36H), 1.13 (s, 3H). $^{13}C$ NMR (100 MHz, $CD_2Cl_2$, 298 K, ppm) δ 155.1, 150.8, 144.3, 143.2, 141.9, 139.5, 138.7, 138.4, 138.0, 137.7, 136.7, 135.8, 134.9, 131.3, 130.8, 129.1, 127.7, 127.3, 126.9, 126.6, 126.6, 126.0, 124.2, 123.6, 121.3, 36.8, 36.2, 34.6, 31.2, 28.6, 26.9, 15.8, 14.0, 13.3. HRMS (MALDI-TOF) calculated for $C_{124}H_{115}BN_2O_2$ $[M+Li]^+$: 1682.9251, found 1682.6324.

**Synthesis of RxFB-2:** To a solution of compound **RxOB-2** (30.0 mg, 0.018 mmol) in anhydrous DCM (5 mL) was added 40 μL concentrated methylsulfonic acid (4 equiv) under -30 °C, the reaction mixture was stirred for

2h. The mixture was washed with water, dried over anhydrous sodium sulfate and evaporated to dryness. The crude compound was used for the next step without further purification. To a solution of the crude product in anhydrous dichloromethane (DCM, 5 mL), triethylamine (0.3 mL) and $BF_3{\cdot}OEt_2$ (0.3 mL, 0.16 mmol) were successively added at -30 °C. The reaction mixture was then allowed to warm to room temperature and stirred overnight. The reaction was quenched by the addition of water, and the aqueous phase was extracted with DCM. The combined organic layers were dried over anhydrous sodium sulfate and concentrated under reduced pressure. The crude product was purified by column chromatography ($SiO_2$, PE/DCM, 2:1) to give ether 10 as a red solid (8 mg, 27%).

$^1$H NMR (600 MHz, $CD_2Cl_2$, , 298 K, ppm) δ 7.69 (s, 2H), 7.62-7.60 (d, $J$ = 12.0 Hz, 8H), 7.53-7.49 (m, 12H), 7.46-7.43 (d, $J$ = 5.8 Hz, 4H), 7.28-7.19 (m, 18H), 7.13-7.12 (m, 8H), 7.04 (s, 4H), 5.37 (s, 2H), 2.27 (s, 3H), 1.80-1.78 (m, 4H), 1.76 (s, 6H), 1.73-1.70 (m, 4H), 1.48 (s, 6H), 1.37 (s, 36H). $^{13}$C NMR (125 MHz, $CD_2Cl_2$, 298 K, ppm) δ 150.7, 149.9, 143.1, 141.8, 139.6, 139.1, 139.0, 138.5, 137.6, 137.1, 135.6, 130.6, 130.0, 127.7, 127.1, 127.0, 126.9, 126.7, 126.5, 126.4, 126.0, 125.9, 125.8, 125.4, 121.7, 35.5, 34.6, 31.3, 29.8, 28.3, 17.0, 13.5. $^{19}$F NMR (377 MHz, DCM-$d_2$) $\delta$ -144.91. HRMS (MALDI-TOF) calculated for $C_{124}H_{117}BF_2N_2O_2$ $[M+Li]^+$: 1722.9375, found 1722.9688.

**Synthesis of Compound 9:** To a 10 mL round bottom flask filled with nitrogen was added **8** (52 mg, 0.1 mmol), $Pd(PPh_3)_2Cl_2$ (7.1 mg, 0.01 mmol), CuI (3.8 mg, 0.02 mmol), dry THF (4.8 mL) and $Et_3N$ (1.2 mL) under nitrogen. The reaction mixture was stirred at room temperature for 5 min and then compound **7** (91.5 mg, 0.3 mmol) in THF (2 mL) was added slowly. The reaction mixture was stirred at room temperature for 24 h. The reaction was quenched with water and the mixture extracted with 3 x 50 mL of dichloromethane. The combined organic layers were dried over sodium sulfate and concentrated in vacuo. Purification by flash chromatography on silica gel (2:1 PE/DCM) afforded compound as red solid (69 mg, 70%).

$^1$H NMR (400 MHz, $CDCl_3$, 298 K, ppm) δ 7.76 (s, 2H), 7.69 (s, 4H), 7.61-7.59 (d, $J$ = 8.0 Hz, 8H), 7.51-7.49 (d, $J$ = 8.0 Hz, 8H), 2.72 (s, 6H), 2.70 (s, 3H) 2.61 (s, 6H), 1.38 (s, 36H). $^{13}$C NMR (100 MHz, $CDCl_3$, 298 K, ppm) δ 142.3, 133.8, 129.5, 127.9, 127.4, 125.9, 124.8, 124.4, 33.6, 30.4, 30.3, 29.2. $^{13}$C NMR (125 MHz, $CDCl_3$, 298 K, ppm) δ 156.9, 150.8, 142.4, 141.9, 137.6, 128.6, 126.9, 126.8, 125.8, 124.1, 116.1, 96.6, 81.8, 34.6, 31.4, 29.7, 19.8, 17.0, 16.3, 16.2, 13.7. HRMS (MALDI-TOF) calculated for $C_{70}H_{73}BF_2N_2$ $[M]^+$: 990.5846, found 990.5999.

**Synthesis of Compound 10:** Compound **9** (40 mg, 0.02 mmol) and Pd/C (40 mg, 10 wt % Pd) were loaded into a pressure reactor and EA (10 mL) and THF (5 mL) and a drop of acetic acid were added. The reaction mixture was stirred under pressure of hydrogen (15 atm) at 40 °C overnight. The mixture was filtered through diatomite and then concentrated. The crude product was purified by flash column chromatography on silica gel eluting with DCM/PE (1/2) to afford compound as red powder (25 mg, 60%).

$^1$H NMR (600 MHz, $CDCl_3$, 298 K, ppm) δ 7.65 (s, 2H), 7.55-7.54 (d, $J$ = 6.0 Hz, 8H), 7.44-7.43(d, $J$ = 6.0 Hz, 8H), 7.30 (s, 4H), 2.82-2.81 (m, 4H), 2.78-2.76 (m, 4H), 2.52 (s, 3H), 2.51 (s, 6H), 2.17 (s, 6H), 1.35 (s, 36H).

$^{13}$C NMR (125 MHz, $CD_2Cl_2$, 298 K, ppm) δ 152.4, 150.4, 142.1, 141.7, 140.3, 138.4, 137.3, 131.9, 129.7, 126.9, 126.9, 126.2, 126.0, 125.7, 125.7, 125.6, 124.5, 124.4, 124.0, 123.8, 123.5, 119.1, 36.9, 34.6, 31.5, 31.5, 31.4, 30.2, 30.2, 29.7, 29.7, 26.4, 22.7, 17.1, 14.4, 14.1, 12.6, 12.6, 12.5. $^{19}$F NMR (377 MHz, DCM-$d_2$) δ -145.5. HRMS (MALDI-TOF) calculated for $C_{70}H_{81}BF_2N_2$ $[M]^+$: 998.6472, found 998.6459.

**Table S1.** Summary of selected spproaches for the synthesis of **RxFB-1**.

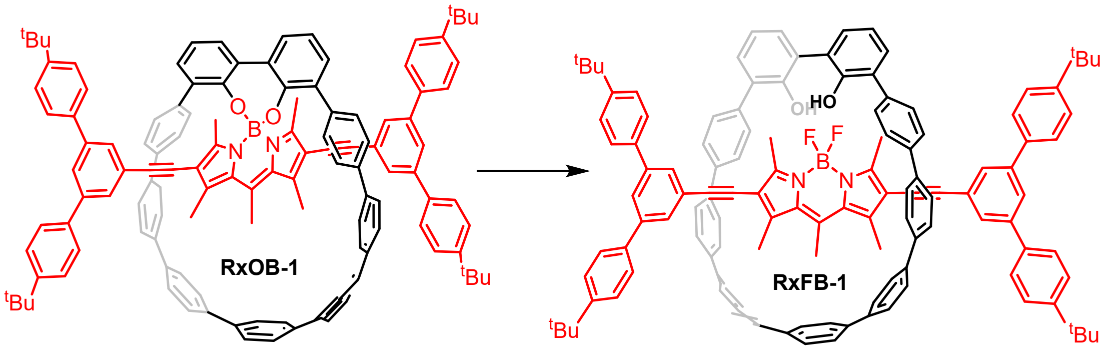


| Entry | Condition | Solution | Temperature | yeild | Reference |
|---|---|---|---|---|---|
| 1 | KOH | MeOH/MeCN | R.T., overnight | No reaction | *Angew.Chem. Int. Ed.*, **2024**, 63, e202318297 |
| | | | Reflux, overnight | No reaction | |
| 2 | $^{t}$BuOK | $^{t}$BuOH | R.T., overnight | No reaction | *J. Org. Chem.*, **2012**, 77, 3439-3453 |
| | | | Reflux, overnight | No reaction | |
| 3 | 500 nm LED | MeOH | R.T., 4h | No reaction | Angew.Chem. Int. Ed., **2018**, 57, 12685-12689 |
| | 530 nm LED | | R.T., 4h | No reaction | |
| 4 | $BCl_3$ | DCM | R.T., overnight | Decomposed | *J. Org. Chem.*, **2021**, 86, 18030-18041 |
| | | | 0 °C, overnight | Decomposed | |
| 5 | $AlCl_3$ | DCM | R.T., overnight | Decomposed | *Eur. J. Inorg. Chem.*, **2020**, 1885-1893 |
| | | | 0 °C, overnight | Decomposed | |
| 6 | $ZrCl_4$ | MeOH/MeCN | R.T., overnight | No reaction | *Eur. J. Org. Chem.*, **2014**, 2105-2110 |
| | | | Reflux, overnight | No reaction | |
| 7 | HCl | MeOH | R.T., overnight | Decomposed | *Dyes Pigments*, **2021**, 193, 1095177 |
| | TFA | MeOH | R.T., overnight | Decomposed | |
| 8 | $MeSO_3H$ | DCM | 40 °C, overnight | Decomposed | *Nat. Synth.*, **2025**, 10.1038/s44160-025-00918-0 |
| | | | R.T., overnight | Decomposed | |
| | | | 0 °C, overnight | Decomposed | |

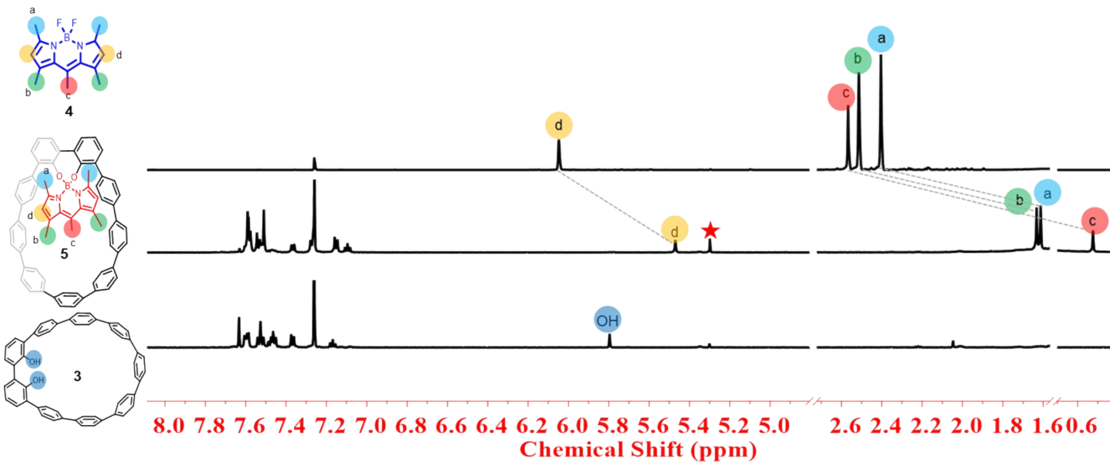


**Figure S1.** Partial $^{1}$H NMR spectra ($CDCl_3$, 298 K) of 3-5. The asterisks indicate solvent peaks.

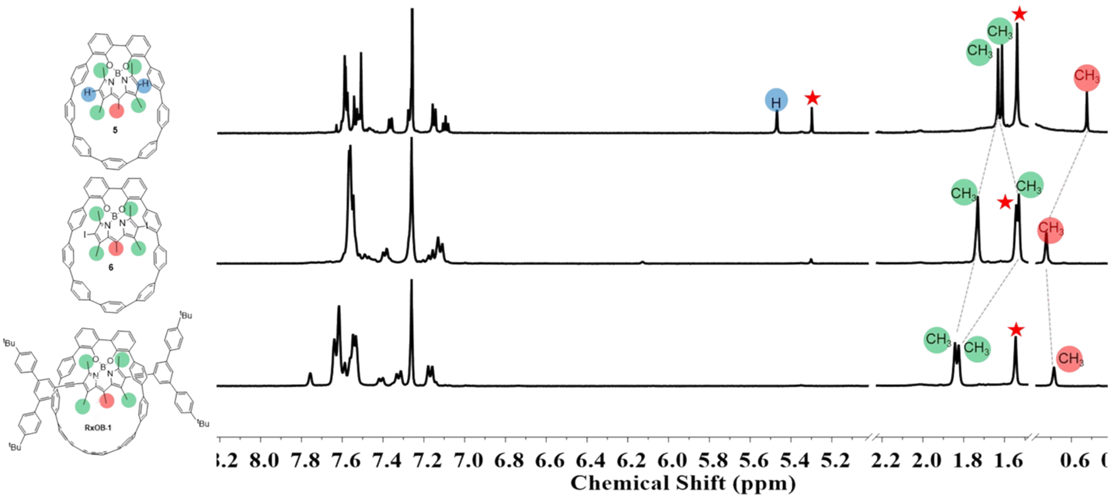


**Figure S2.** Partial $^{1}H$ NMR spectra ($CDCl_3$, 298 K) of **5**, **6**, and **RxOB-1**. The asterisks indicate solvent/water peaks.

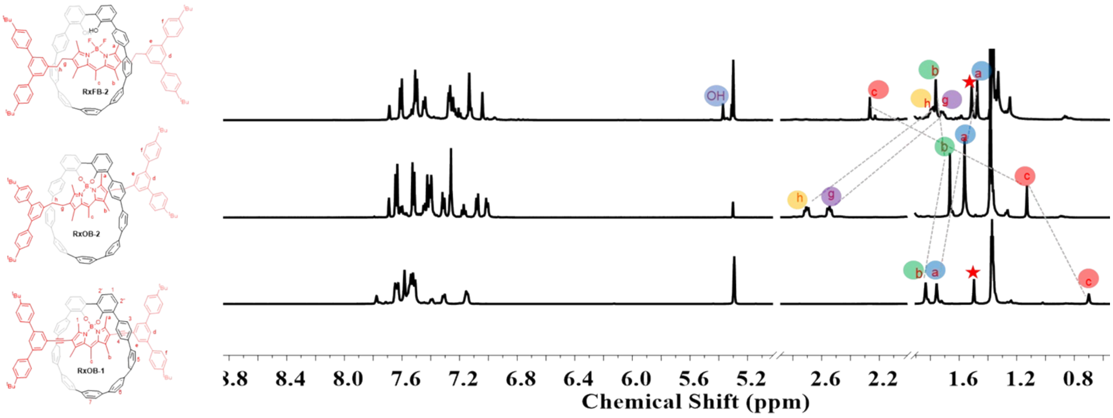


**Figure S3.** Partial $^{1}H$ NMR spectra($CD_2Cl_2$, 298 K ) of **RxOB-1**, **RxOB-2** and **RxFB-2**. The asterisks indicate solvent/water peaks.

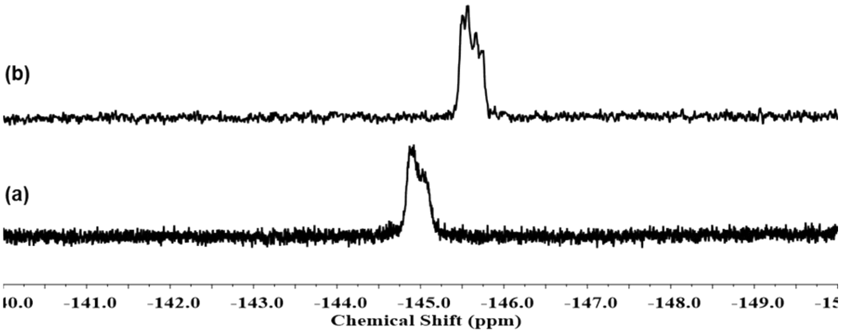


**Figure S4.** $^{9}F$ NMR (377 MHz, DCM-$d_2$) of (a) **10** and (b) **RxFB-2**.

**Table S2.** Chemical shifts of different compound.

| Compound | Ha (δ ppm) | Hb (δ ppm) | Hc (δ ppm) |
|---|---|---|---|

| | | | |
|---|---|---|---|
| **4 (in $CDCl_3$)** | 2.40 | 2.51 | 2.57 |
| **5 (in $CDCl_3$)** | 1.61 | 1.63 | 0.52 |
| **6 (in $CDCl_3$)** | 1.53 | 1.73 | 0.72 |
| **RxOB-1 (in $CDCl_3$)** | 1.82 | 1.84 | 0.69 |
| **RxOB-1 (in $CD_2Cl_2$)** | 1.76 | 1.83 | 0.70 |
| **RxOB-2 (in $CD_2Cl_2$)** | 1.56 | 1.66 | 1.13 |
| **RxFB-2 (in $CD_2Cl_2$)** | 1.48 | 1.78 | 2.27 |
| **9 (in $CD_2Cl_2$)** | 2.62 | 2.67 | 2.71 |
| **10 (in $CD_2Cl_2$)** | 2.20 | 2.39 | 2.53 |

## 3. X-ray Crystallography

Single crystal X-ray diffraction data were collected on a Bruker D8 Venture . The crystal was kept at 170 K during data collection. Using Olex2, the structure was solved with the ShelXT structure solution program using Intrinsic Phasing and refined with the ShelXL refinement package using Least Squares minimization. The disordered solvent molecules were removed with the SQUEEZE routine in PLATON and the solvent-free model was employed for the final refinement. All non-hydrogen atoms were refined anisotropically. All hydrogen atoms were positioned by geometric idealization. Details of the crystal data and a summary of the intensity data collection parameters for them, are listed in Table S3.

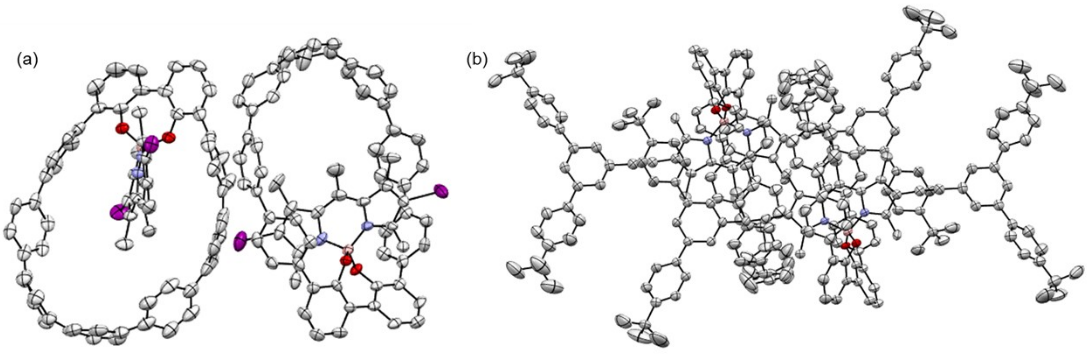


**Figure S5.** X-ray crystal structures of (a) **6** and (b) **RxOB-1** with 50% probability thermal ellipsoids.

**Table S3.** Crystal data and structure refinement for compound **6** and **RxOB-1**.

| **Compounds** | **6** | **RxOB-1** |
|---|---|---|
| **Identification code** | 2516995 | 2516996 |
| **Empirical formula** | $C_{139}H_{103}B_2Cl_9I_4N_4O_4$ | $C_{131}H_{121}BCl_{14}N_2O_2$ |
| **Formula weight** | 2741.52 | 2262.40 |
| **Temperature/K** | 170 | 170 |
| **Crystal system** | triclinic | triclinic |
| **Space group** | P-1 | P-1 |
| **a/Å** | 16.2128(13) | 15.0856(4) |
| **b/Å** | 17.9501(16) | 19.0199(6) |
| **c/Å** | 26.561(2) | 24.4989(7) |
| **α/°** | 70.701(2) | 99.458(2) |
| **β/°** | 76.903(2) | 98.306(2) |
| **γ/°** | 69.938(2) | 110.659(2) |
| **Volume/Å$^3$** | 6796.8(10) | 6330.0(3) |
| **Z** | 2 | 2 |
| **$\rho_{calc}$g/cm$^3$** | 1.340 | 1.187 |
| **$\mu$/mm$^{-1}$** | 1.145 | 3.166 |
| **F(000)** | 2744.0 | 2360.0 |
| **Crystal size/mm$^3$** | 0.12×0.06×0.05 | 0.08×0.04×0.02 |
| **Radiation** | MoKα (λ=0.71073) | CuKα (λ=1.54178) |
| **2Θ range for data collection/°** | 3.966 to 53.112 | 5.106 to 127.712 |

| | | |
|---|---|---|
| **Index ranges** | -20≤h≤20, -22≤k≤22, -33≤l≤32 | -16≤h≤17, -21≤k≤22, -27≤l≤28 |
| **Reflections collected** | 76093 | 59072 |
| **Independent reflections** | 27897 [$R_{int}$=0.0777, $R_{sigma}$=0.0992] | 20716 [$R_{int}$=0.0700, $R_{sigma}$=0.0694] |
| **Data/restraints/parameters** | 27897/6/1469 | 20716/51/1210 |
| **Goodness-of-fit on $F^2$** | 1.042 | 1.061 |
| **Final R indexes [I>=2σ (I)]** | $R_1$=0.0924, $wR_2$=0.2443 | $R_1$=0.0742, $wR_2$=0.2116 |
| **Final R indexes [all data]** | $R_1$=0.1442, $wR_2$=0.2799 | $R_1$=0.0987, $wR_2$ = 0.2301 |
| **Largest diff. peak/hole/e Å$^{-3}$** | 4.84/-2.60 | 0.34/-0.28 |

## 4. Photophysical Properties

The energy transfer efficiency ($\Phi_{ET}$), which indicating the fraction of the absorbed energy transferred to the acceptor, is experimentally measured as a ratio of the fluorescence intensities of the donor in the absence and presence of the acceptor ($I_D$ and $I_{DA}$) by the equation S1.

$$\Phi_{ET} = 1 - \frac{I_{DA}}{I_A}$$ ……S1

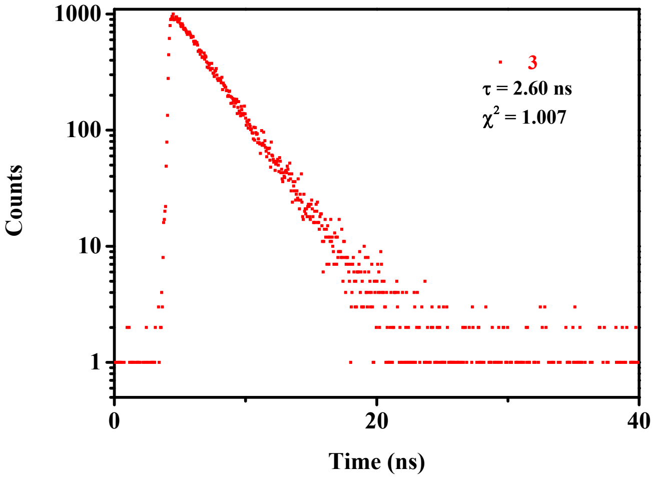


**Figure S6.** Emission lifetime of **3** in $CH_2Cl_2$ at 25 °C.

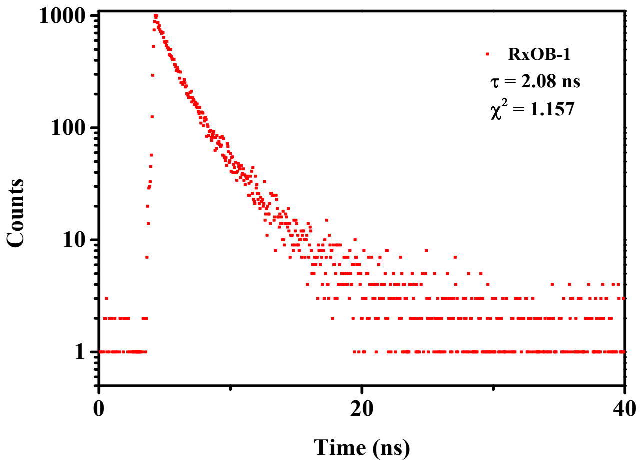


**Figure S7.** Emission lifetime of **RxOB-1** in $CH_2Cl_2$ at 25 °C.

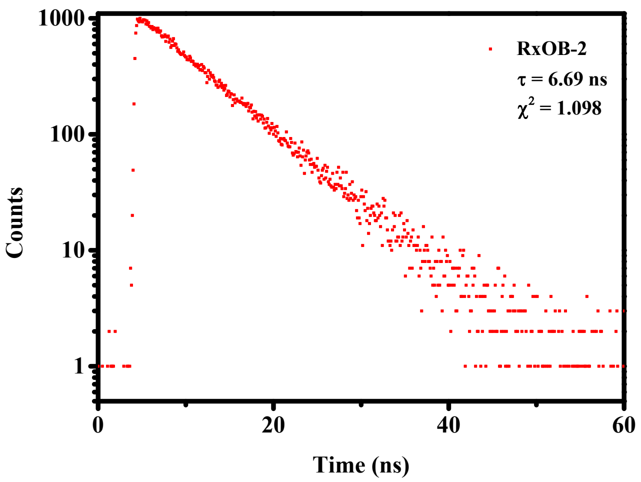


**Figure S8.** Emission lifetime of **RxOB-2** in $CH_2Cl_2$ at 25 °C.

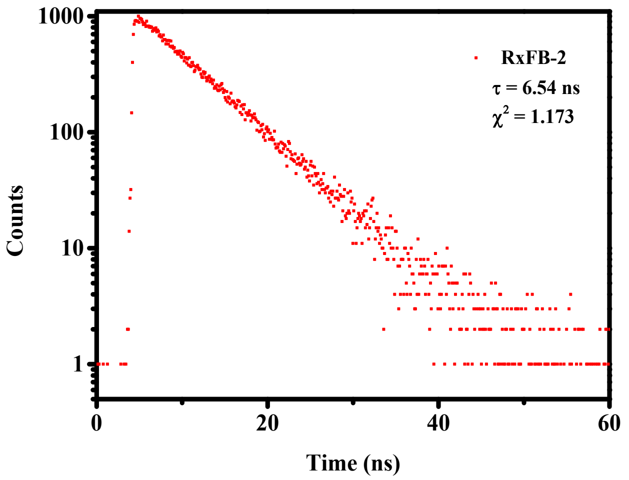


**Figure S9.** Emission lifetime of **RxFB-2** in $CH_2Cl_2$ at 25 °C.

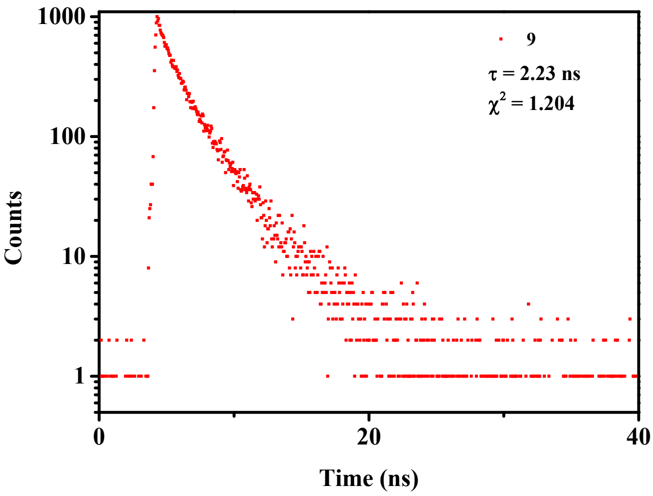


**Figure S10.** Emission lifetime of **9** in $CH_2Cl_2$ at 25 °C.

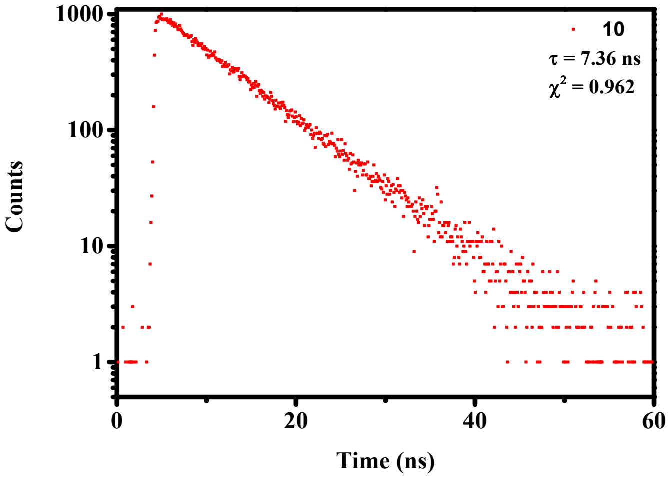


**Figure S11.** Emission lifetime of **10** in $CH_2Cl_2$ at 25 °C.

## 5. $^1H$, $^{13}C$ NMR and Ms Spectra

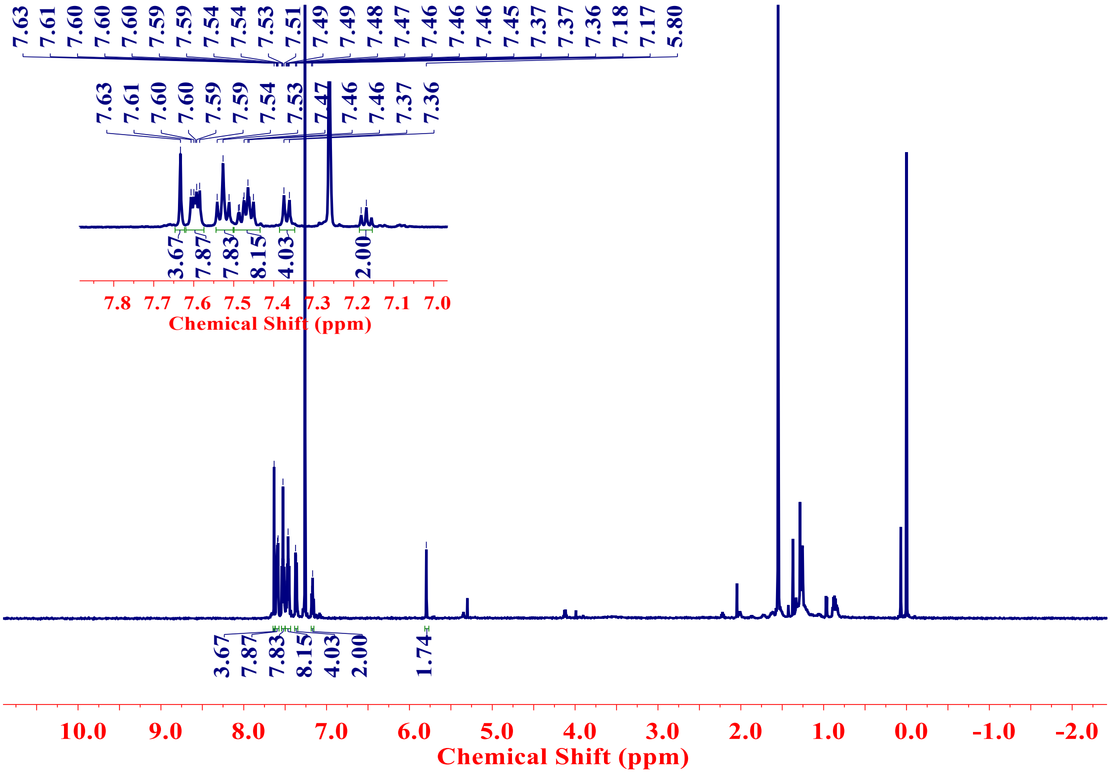

**Figure S12.** $^1H$ NMR of compound **3** (600 MHz, $CDCl_3$).

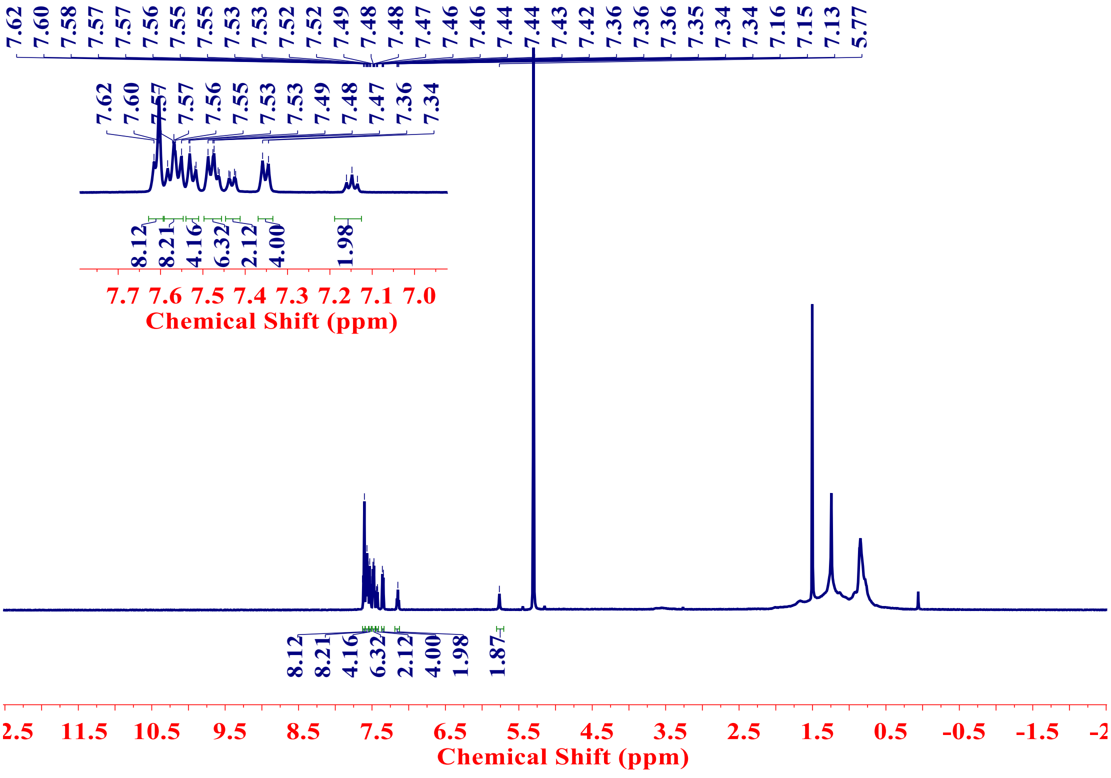

**Figure S13.** $^1H$ NMR of compound **3** (600 MHz, $CD_2Cl_2$).

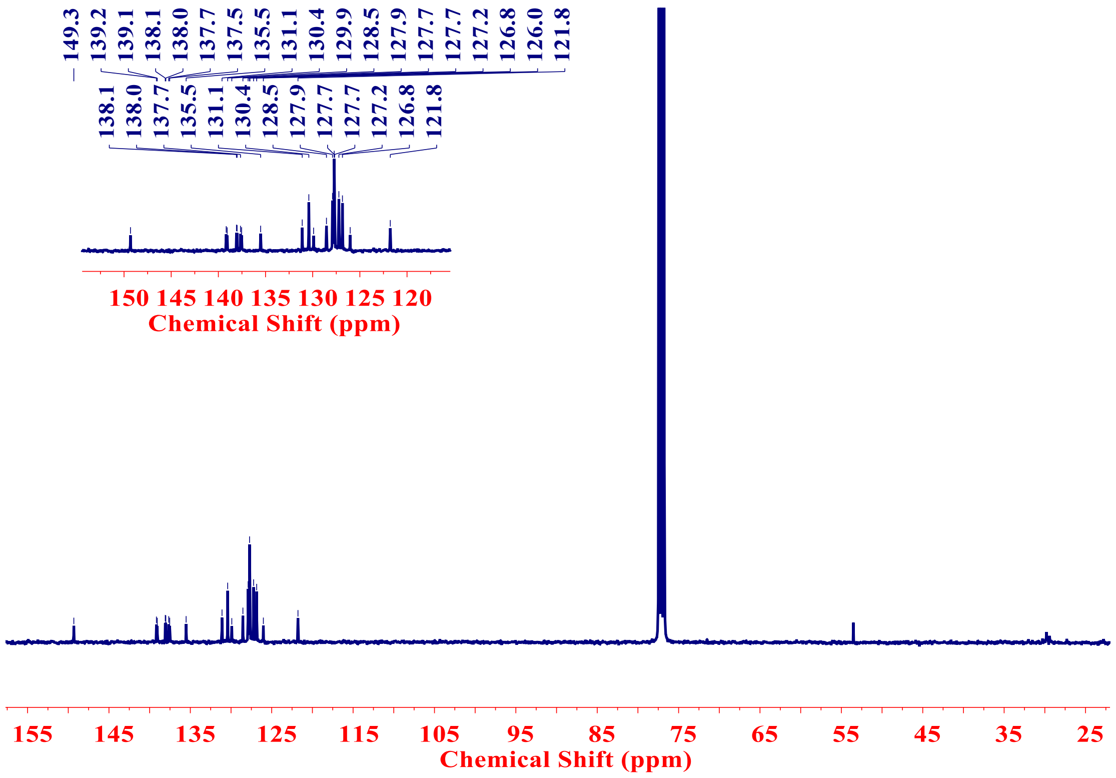


**Figure S14.** $^{13}C$ NMR of compound **3** (100 MHz, $CDCl_3$).

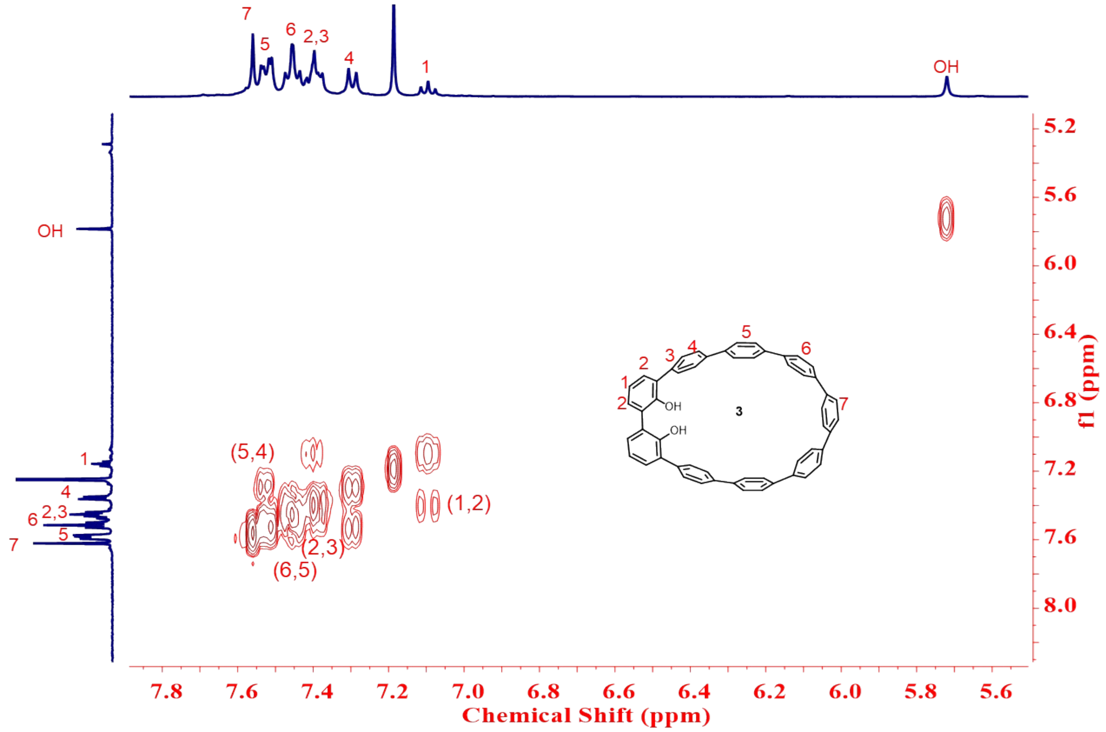


**Figure S15.** $^1H$−$^1H$ COSY spectrum ($CDCl_3$, 400 MHz, 298 K) of compound **3**.

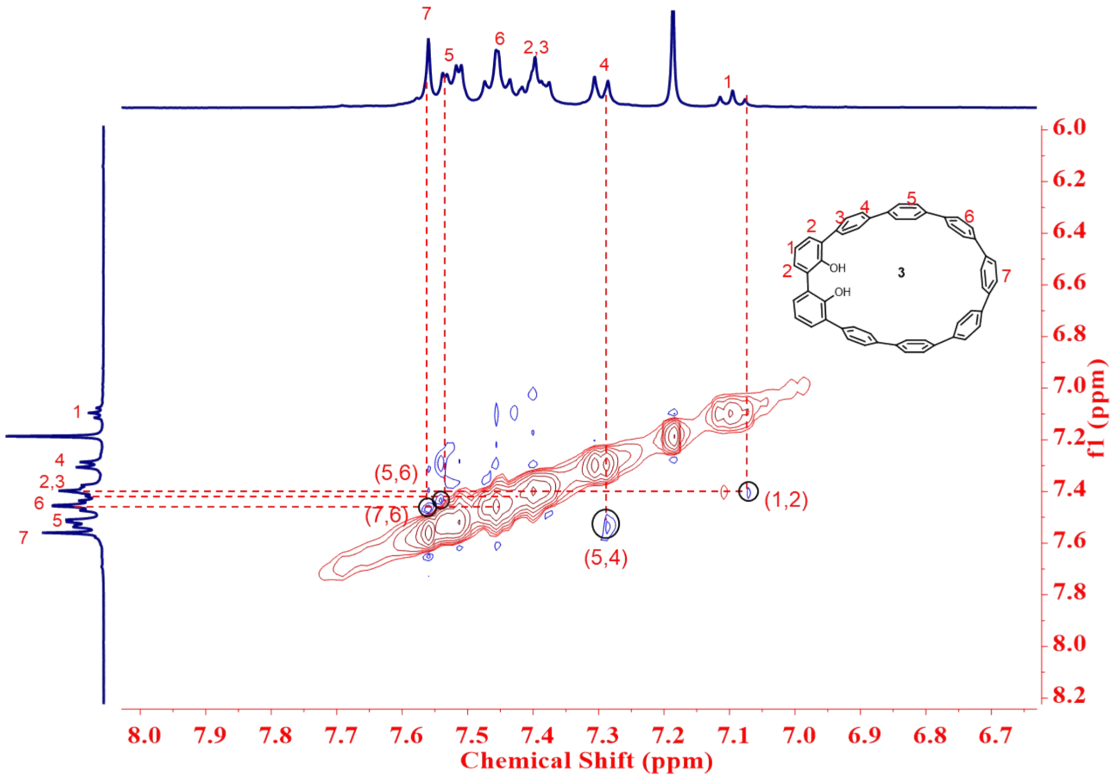


**Figure S16.** Partial 2D-NOESY spectrum ($CDCl_3$, 400 MHz, 298 K) of compound **3**.

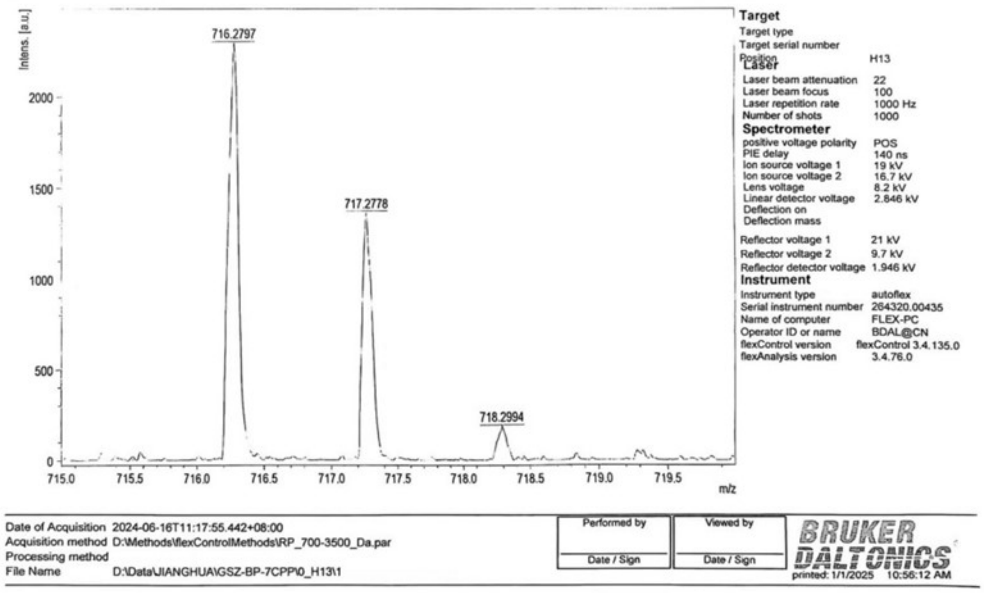


**Figure S17.** MS spectrum of **3**.

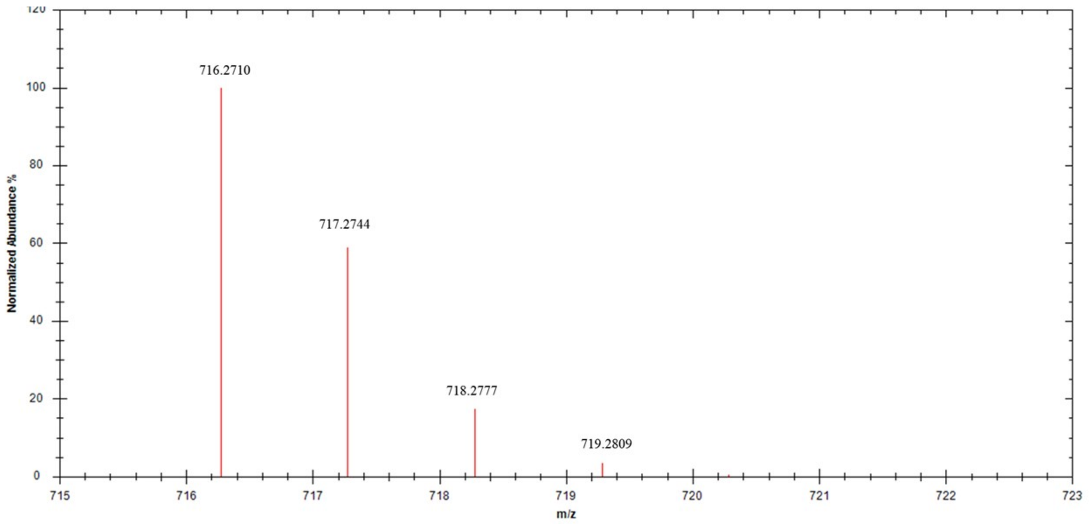


**Figure S18.** Simulated HR-MS spectrum of 3.

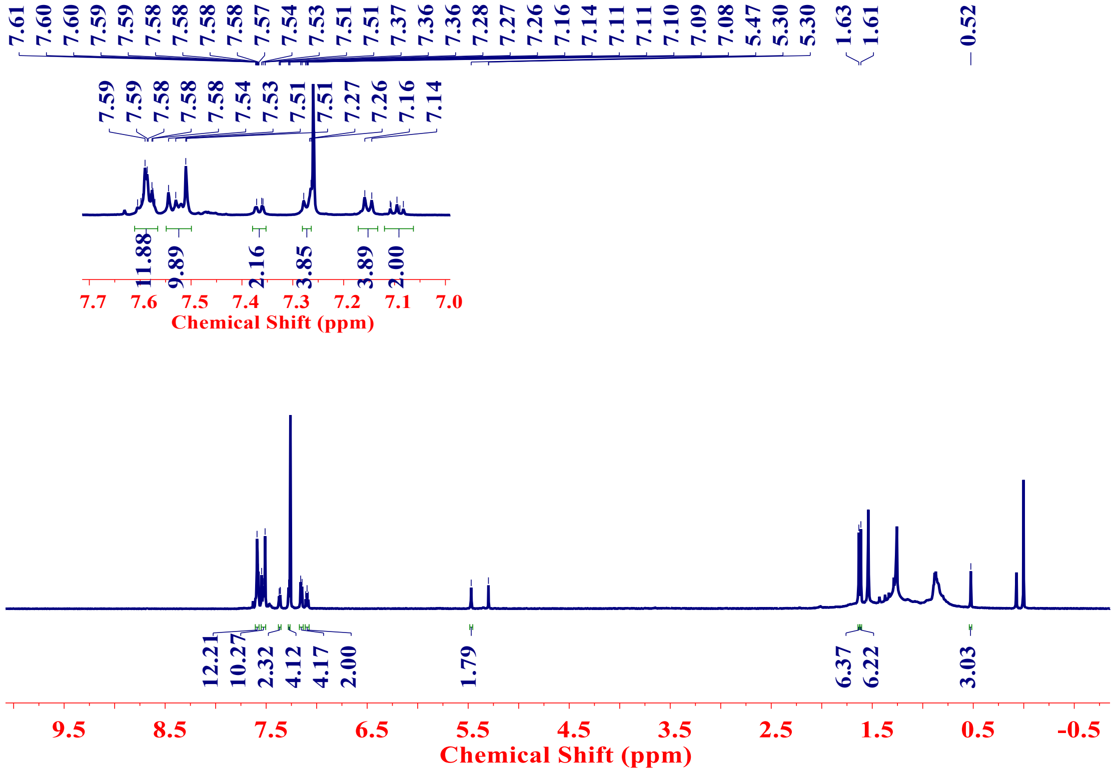


**Figure S19.** $^{1}H$ NMR of compound **5** (600 MHz, $CDCl_3$).

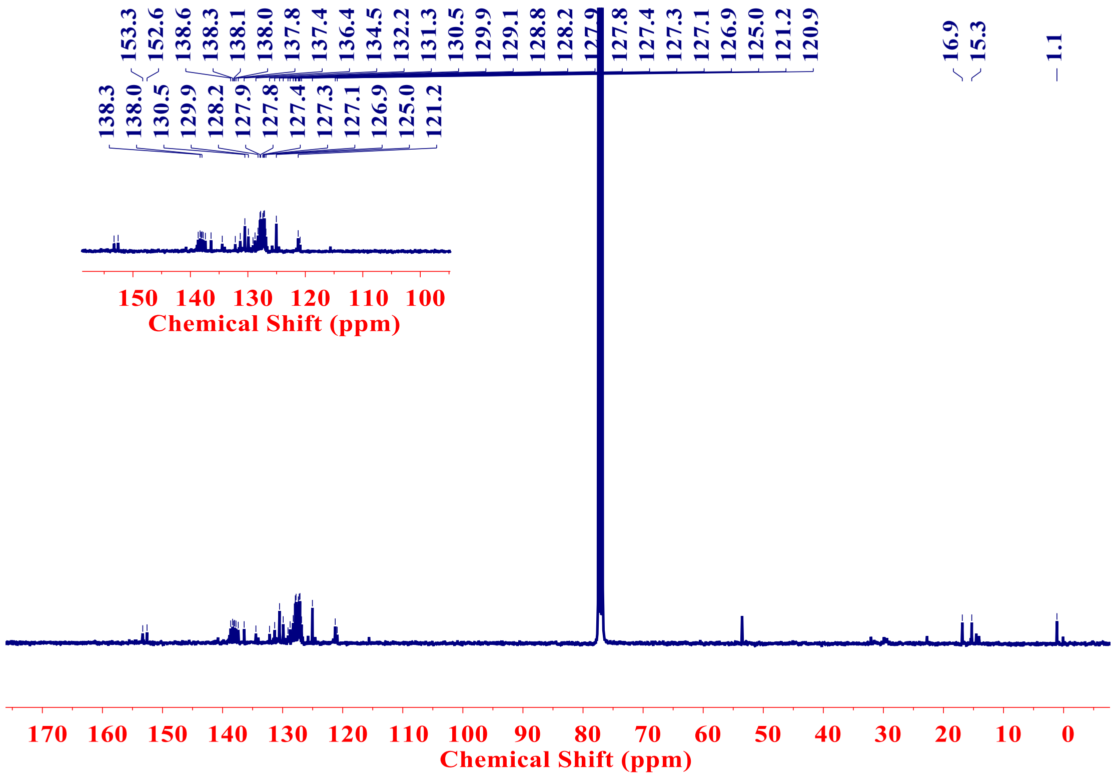


**Figure S20.** $^{13}C$ NMR of compound **5** (100 MHz, $CDCl_3$).

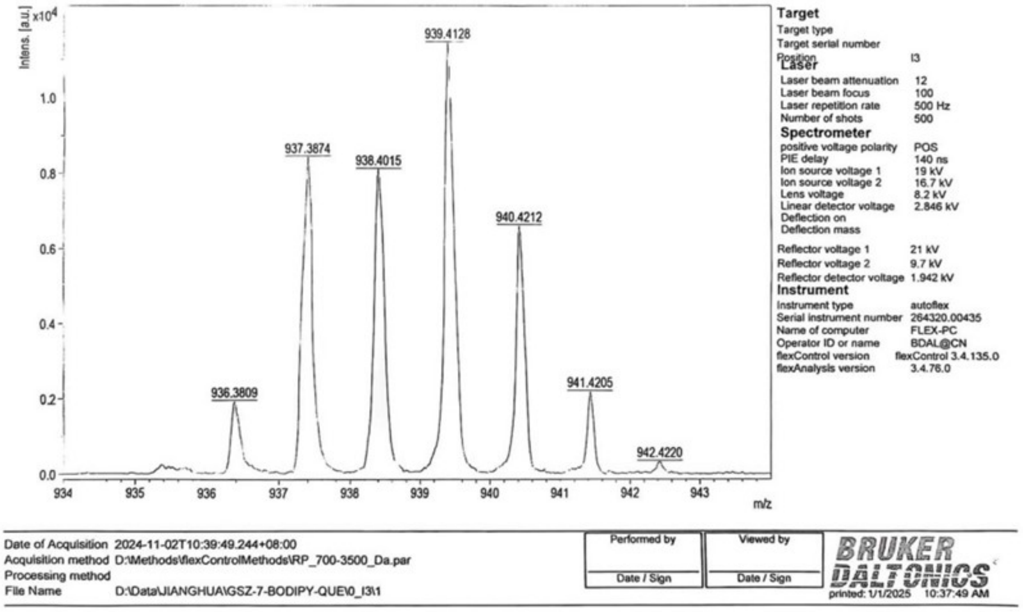


**Figure S21.** MS spectrum of **5**.

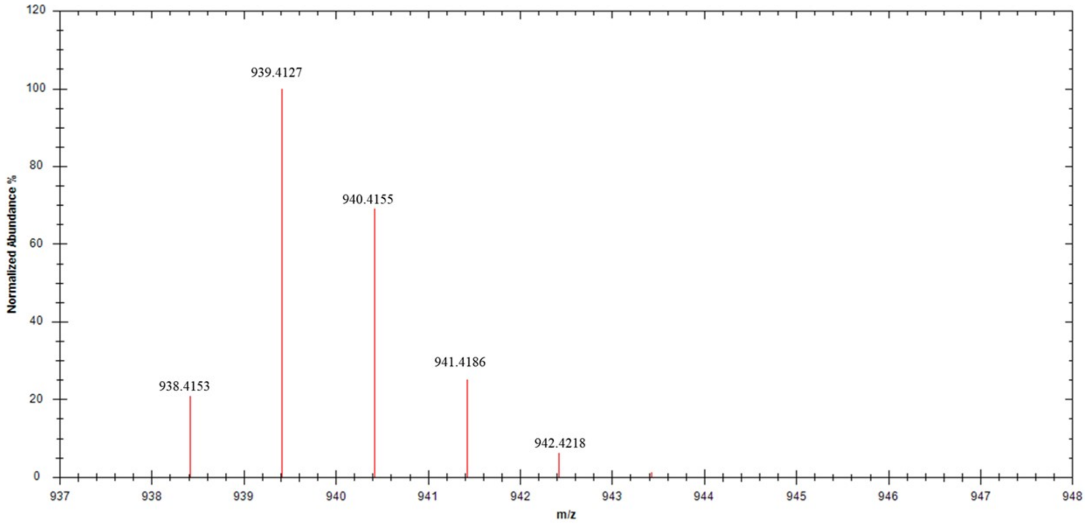


**Figure S22**. Simulated HR-MS spectrum of 5.

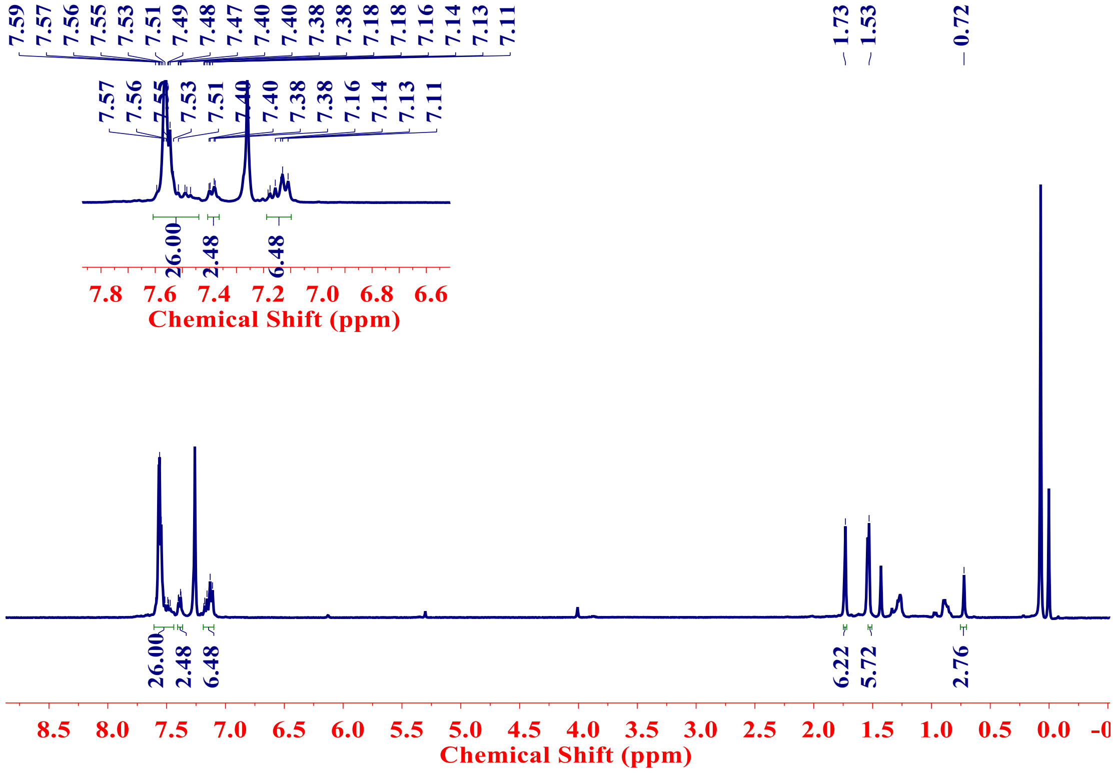


**Figure S23.** $^{1}H$ NMR of compound **6** (600 MHz, $CDCl_3$).

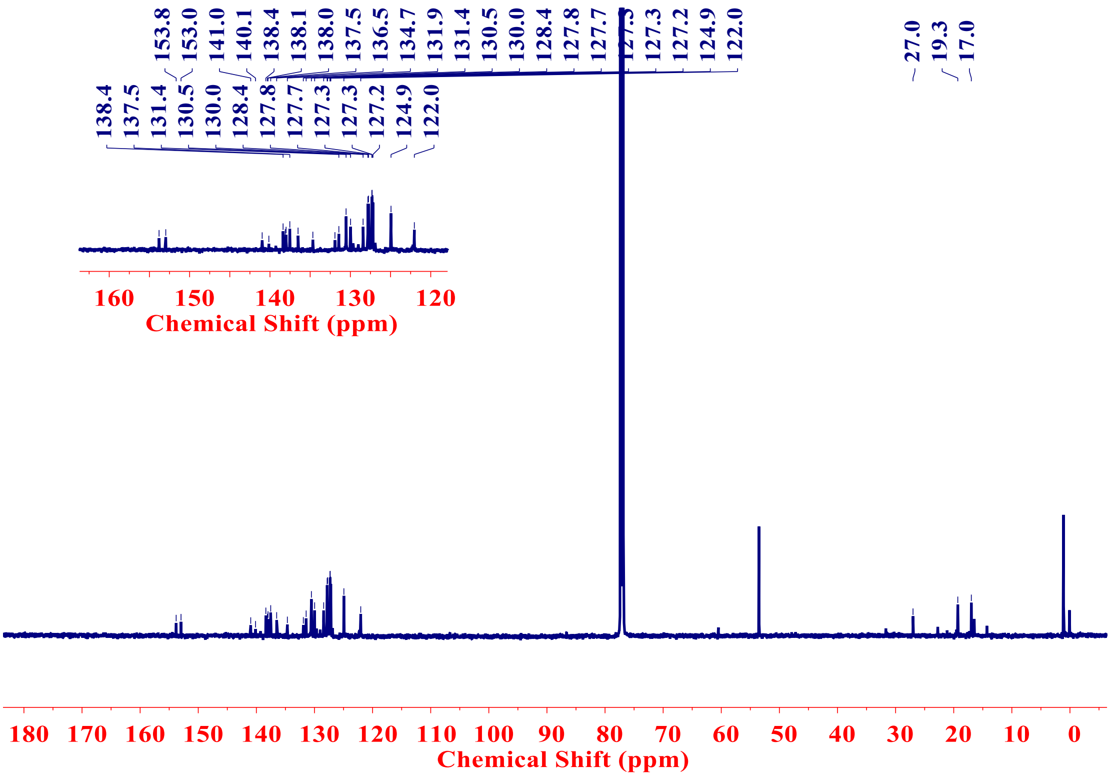


**Figure S24.** $^{13}C$ NMR of compound **6** (150 MHz, $CDCl_3$).

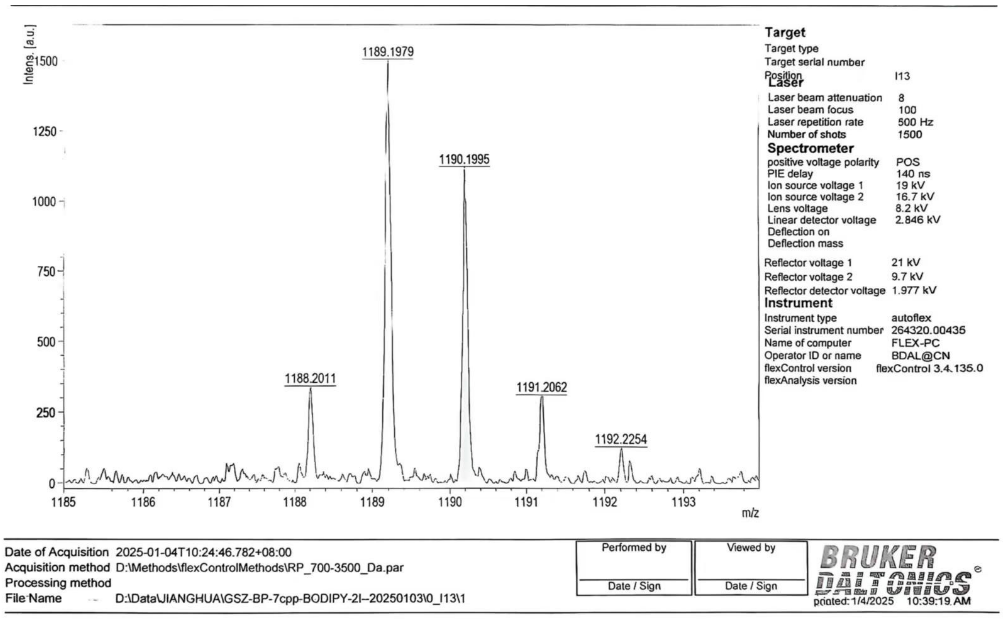


**Figure S25.** MS spectrum of **6.**

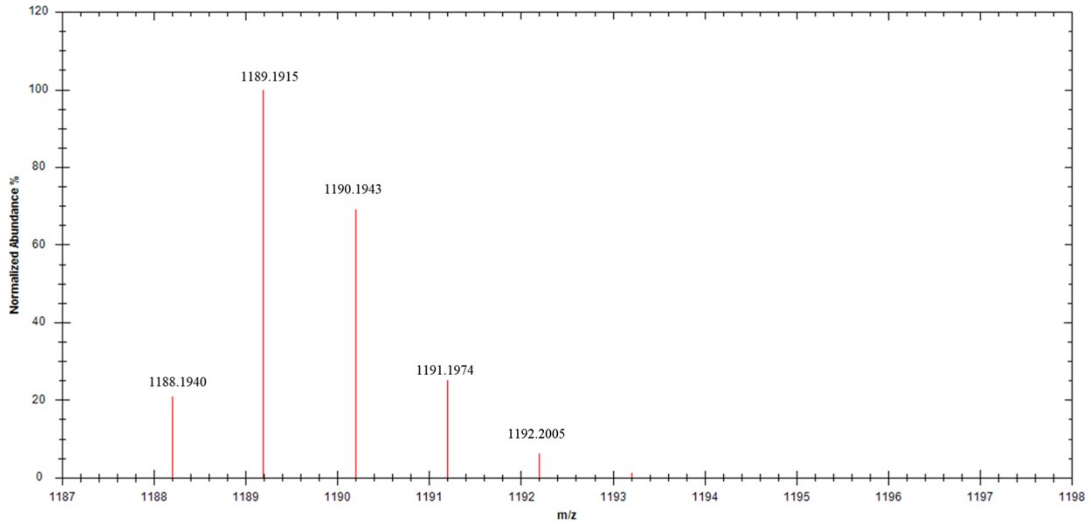


**Figure S26**. Simulated HR-MS spectrum of 6.

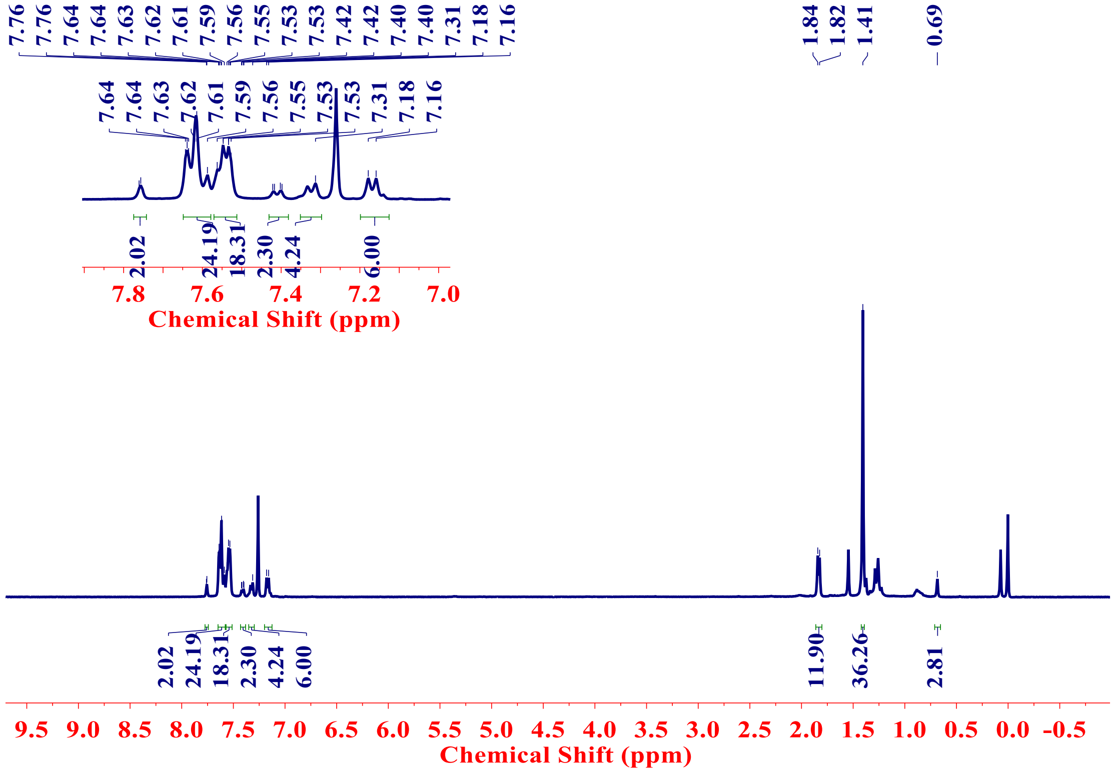


**Figure S27.** $^{1}$H NMR of **RxOB-1** (400 MHz, $CDCl_3$).

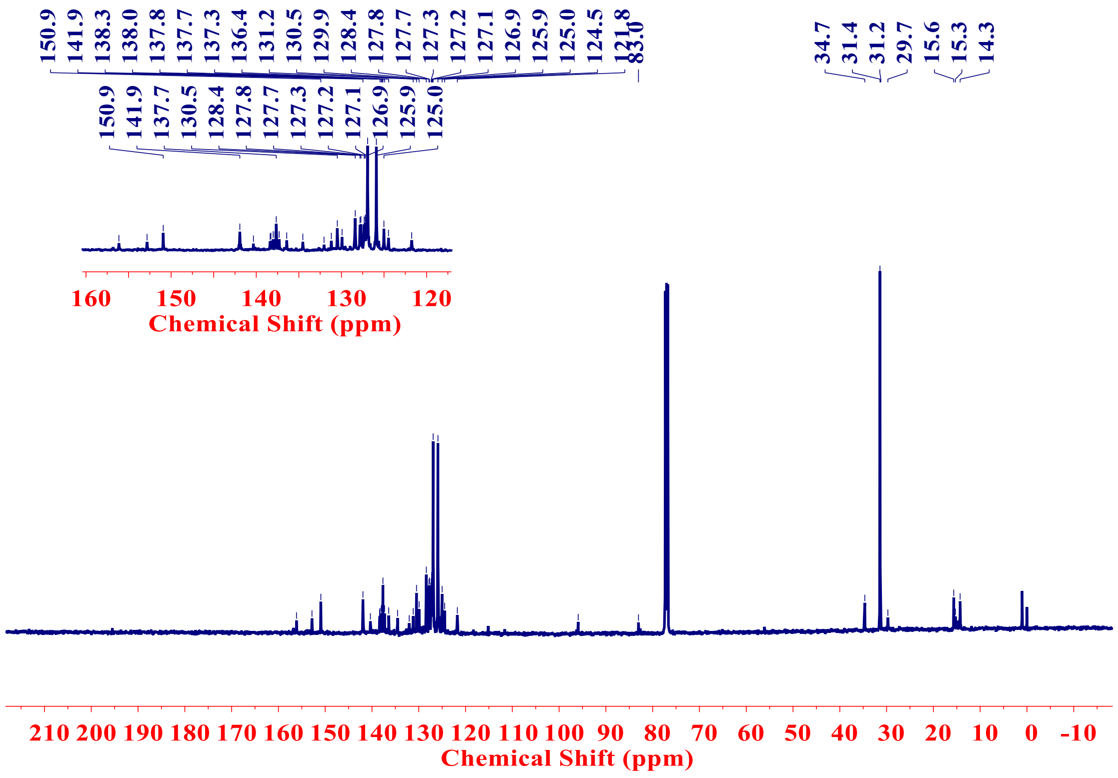


**Figure S28.** $^{13}C$ NMR of **RxOB-1** (125 MHz, $CDCl_3$).

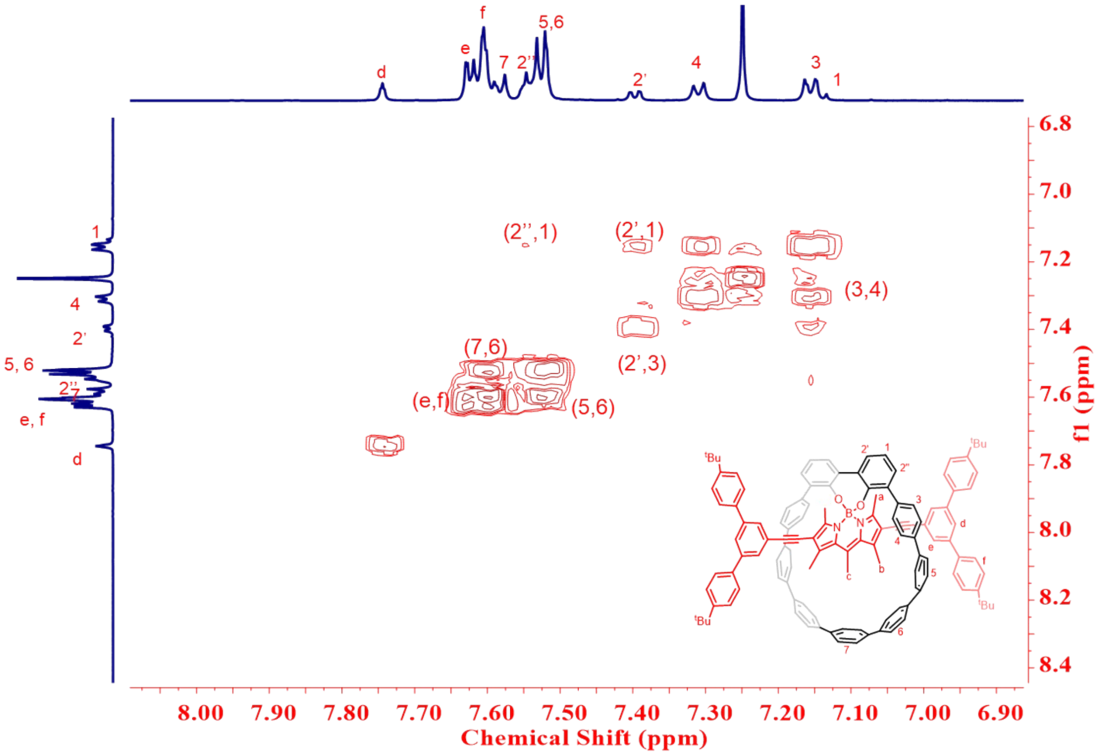


**Figure S29**. $^1H$–$^1H$ COSY spectrum ($CDCl_3$, 400 MHz, 298 K) of **RxOB-1**.

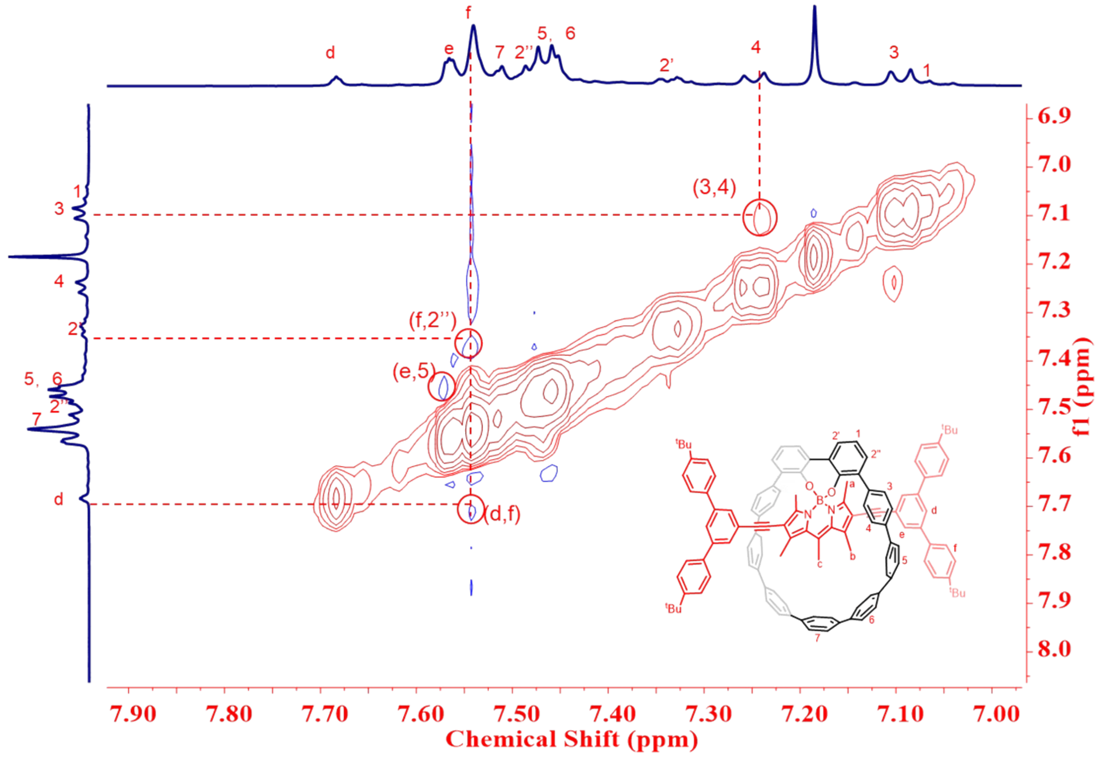


**Figure S30.** Partial 2D-NOESY spectrum ($CDCl_3$, 400 MHz, 298 K) of **RxOB-1**.

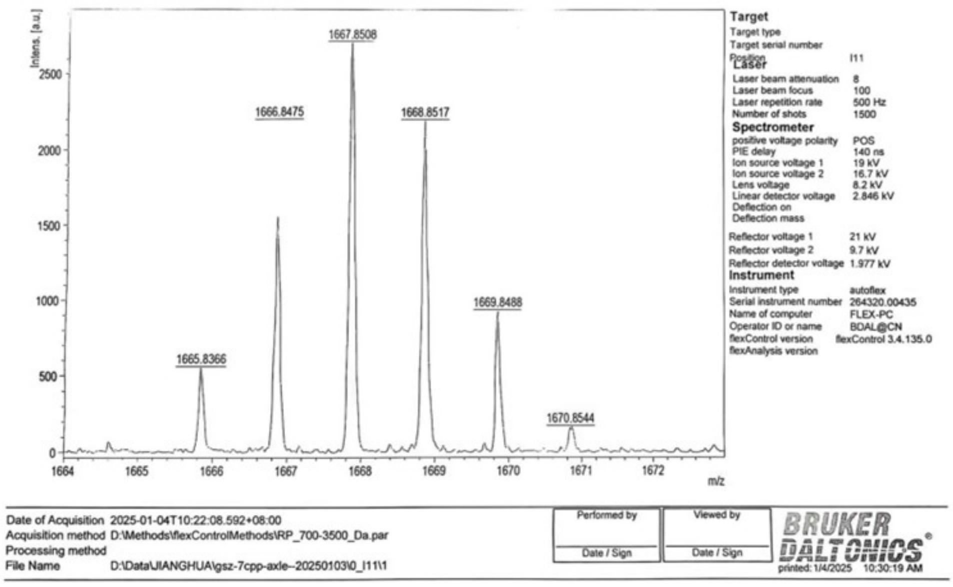


**Figure S31.** MS spectrum of **RxOB-1**.

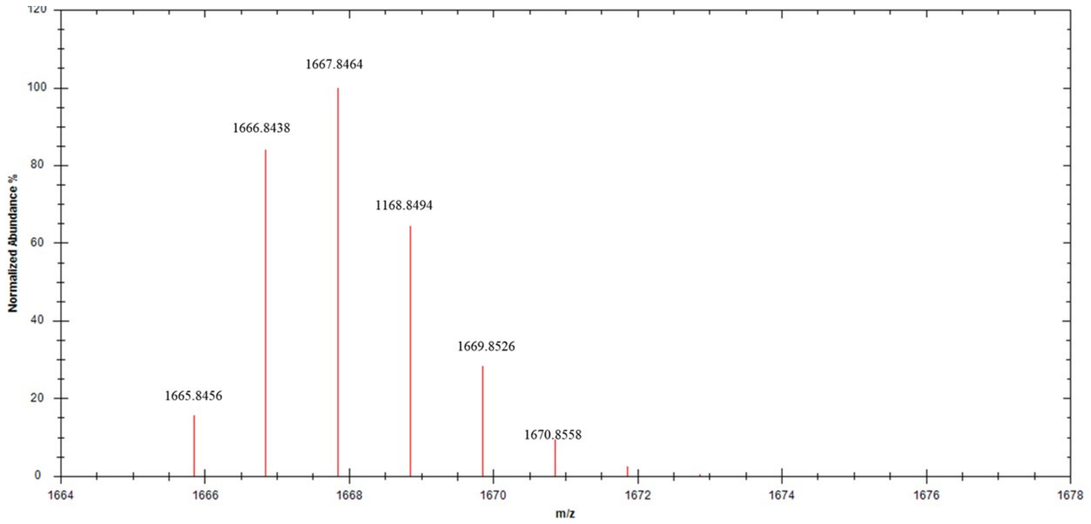


**Figure S32**. Simulated HR-MS spectrum of **RxOB-1**.

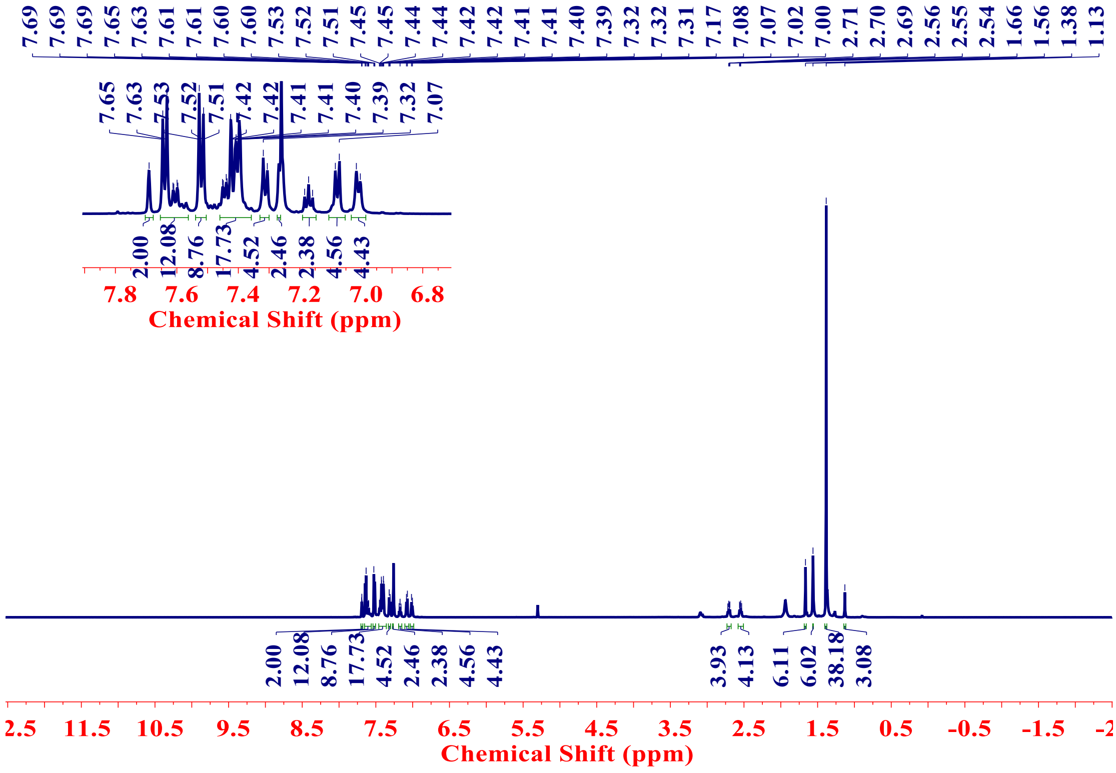


**Figure S34.** $^{1}$H NMR of **RxOB-2** (600 MHz, $CDCl_3$).

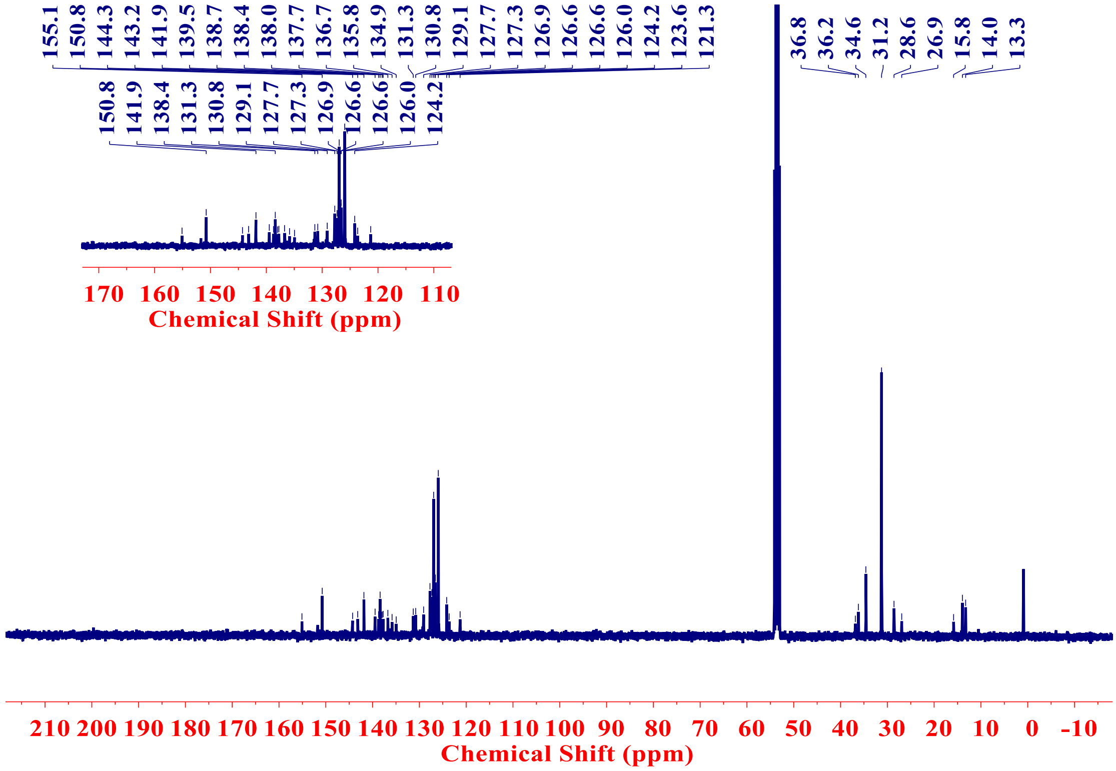


**Figure S35.** $^{13}C$ NMR of **RxOB-2** (100 MHz, $CD_2Cl_2$).

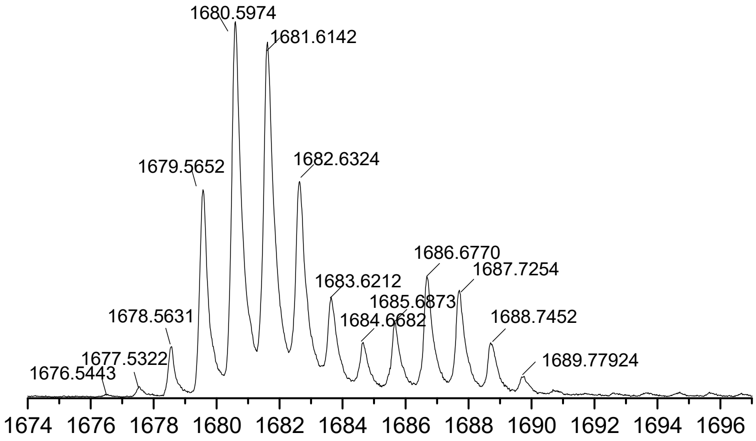


**Figure S36.** MS spectrum of **RxOB-2**.

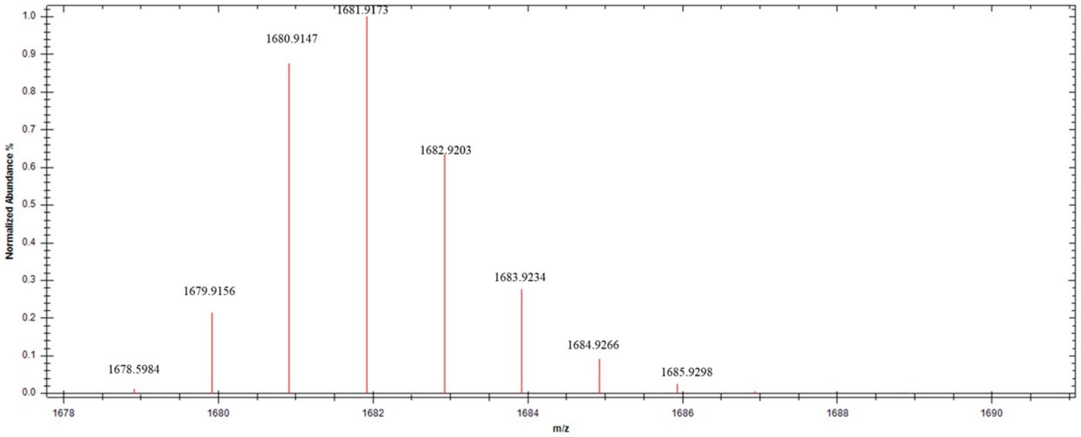


**Figure S37**. Simulated HR-MS spectrum of **RxOB-2**.

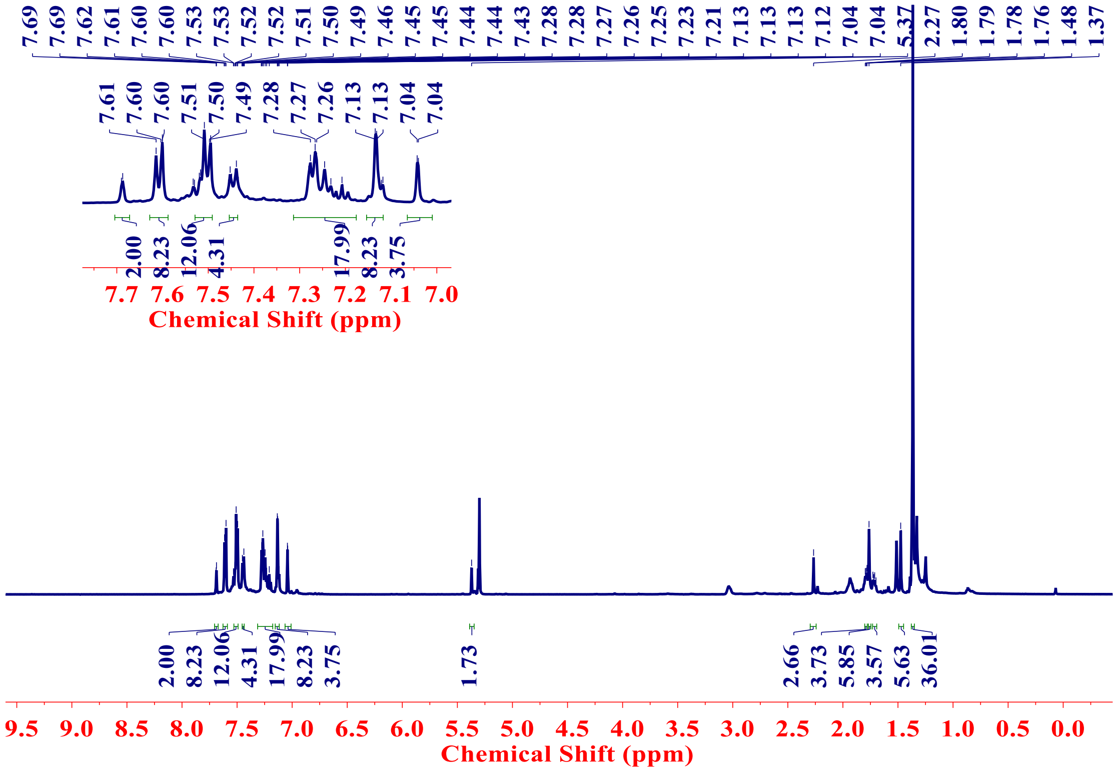


**Figure S38.** $^{1}H$ NMR of **RxFB-2** (600 MHz, $CD_2Cl_2$).

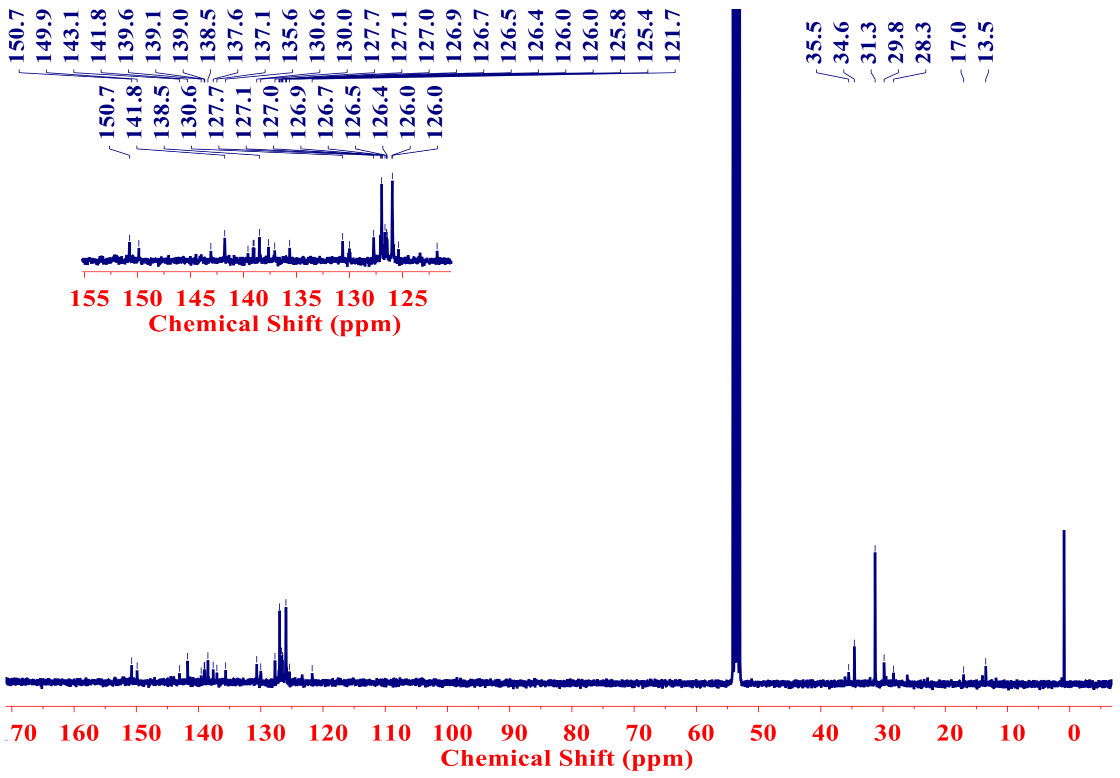


**Figure S39.** $^{13}C$ NMR of **RxFB-2** (125 MHz, $CD_2Cl_2$).

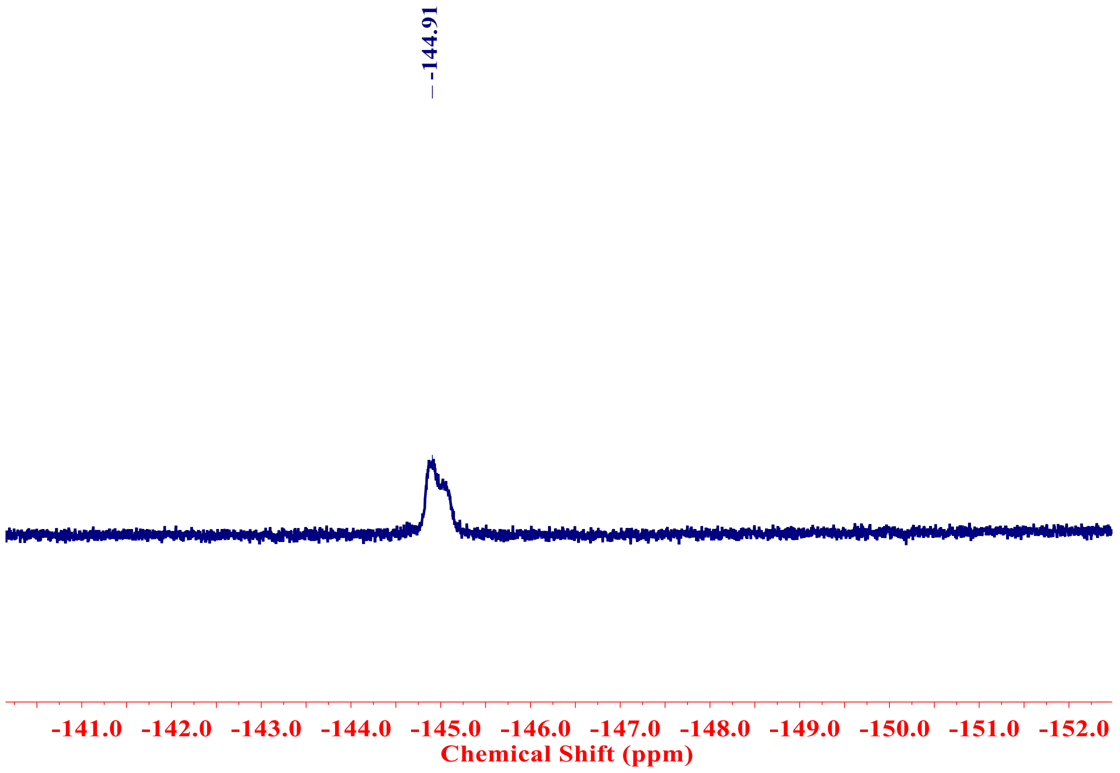


**Figure S40.** $^{19}F$ NMR spectrum of **RxFB-2** (377 MHz, $CD_2Cl_2$).

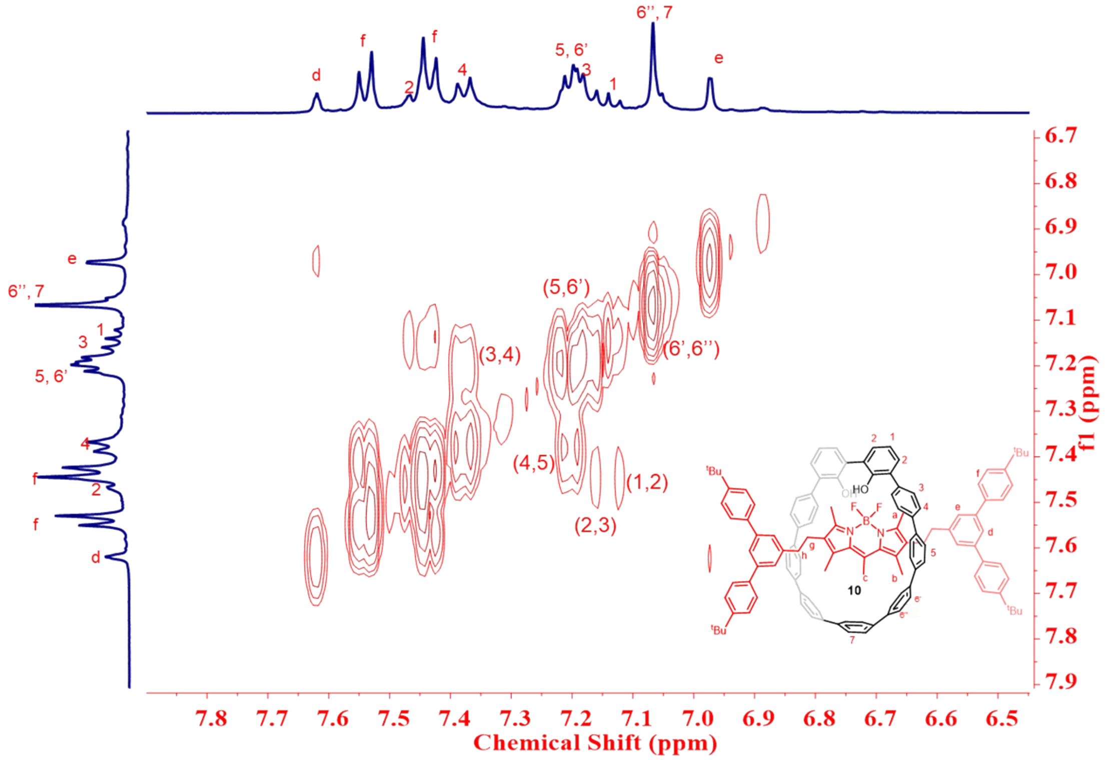


**Figure S41.** $^{1}H-^{1}H$ COSY spectrum ($CDCl_3$, 400 MHz, 298 K) of **RxFB-2**.

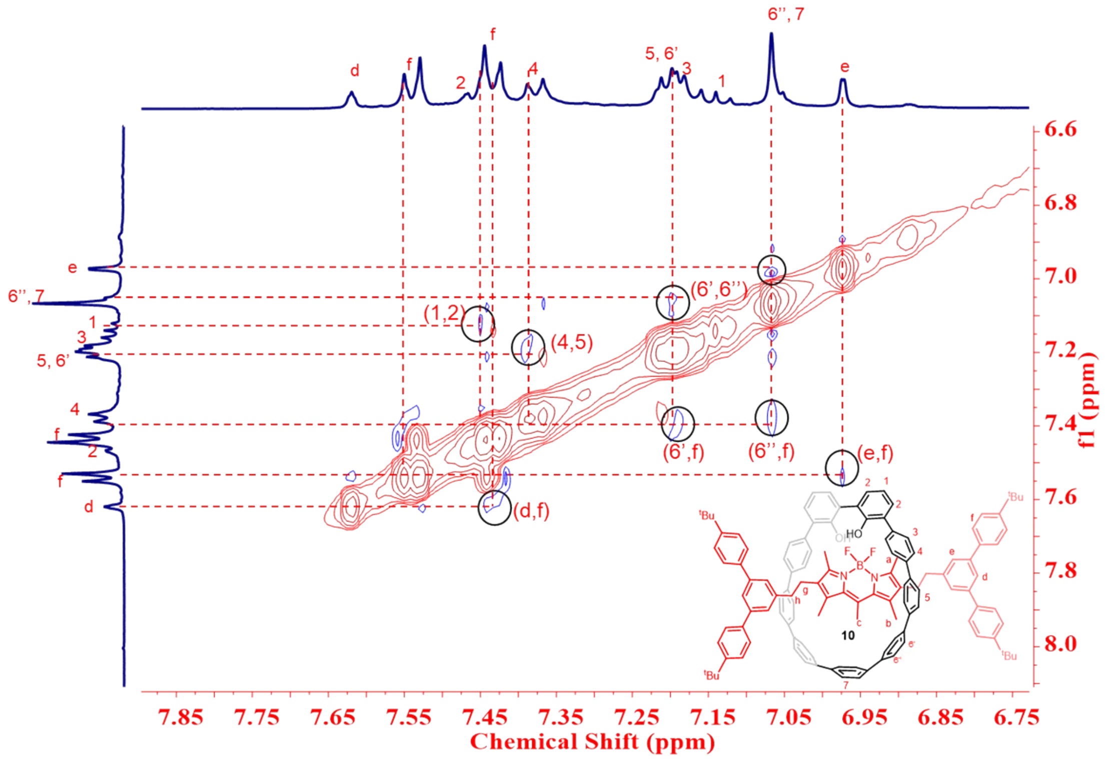


**Figure S42.** Partial 2D-NOESY spectrum ($CDCl_3$, 400 MHz, 298 K) of **RxFB-2**.

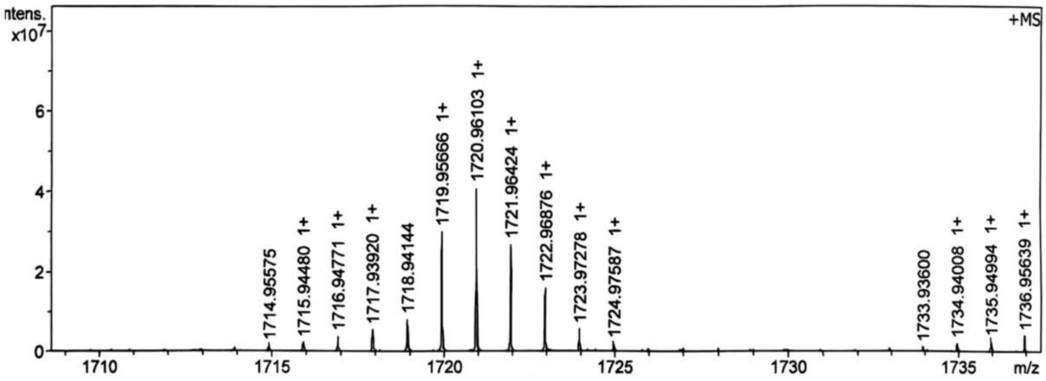


**Figure S43.** MS spectrum of **RxFB-2**

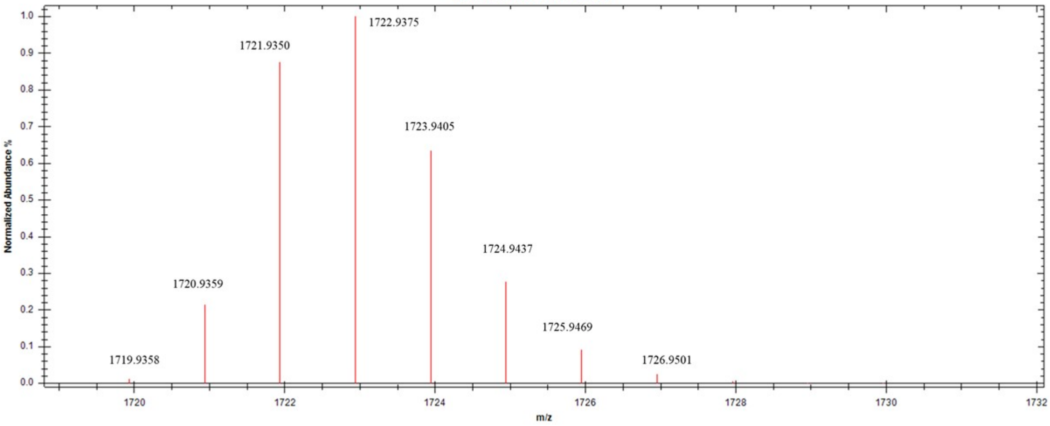


**Figure S44**. Simulated HR-MS spectrum of **RxFB-2**.

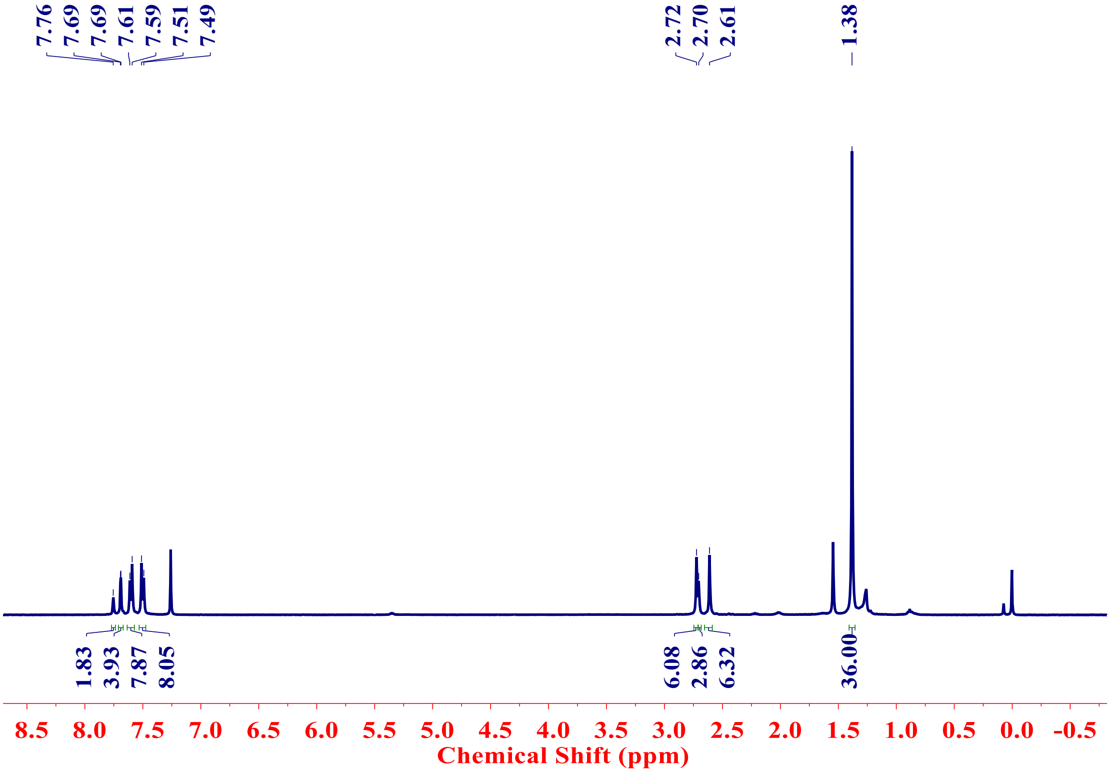


**Figure S45.** $^{1}$H NMR of compound **9** (400 MHz, $CDCl_3$).

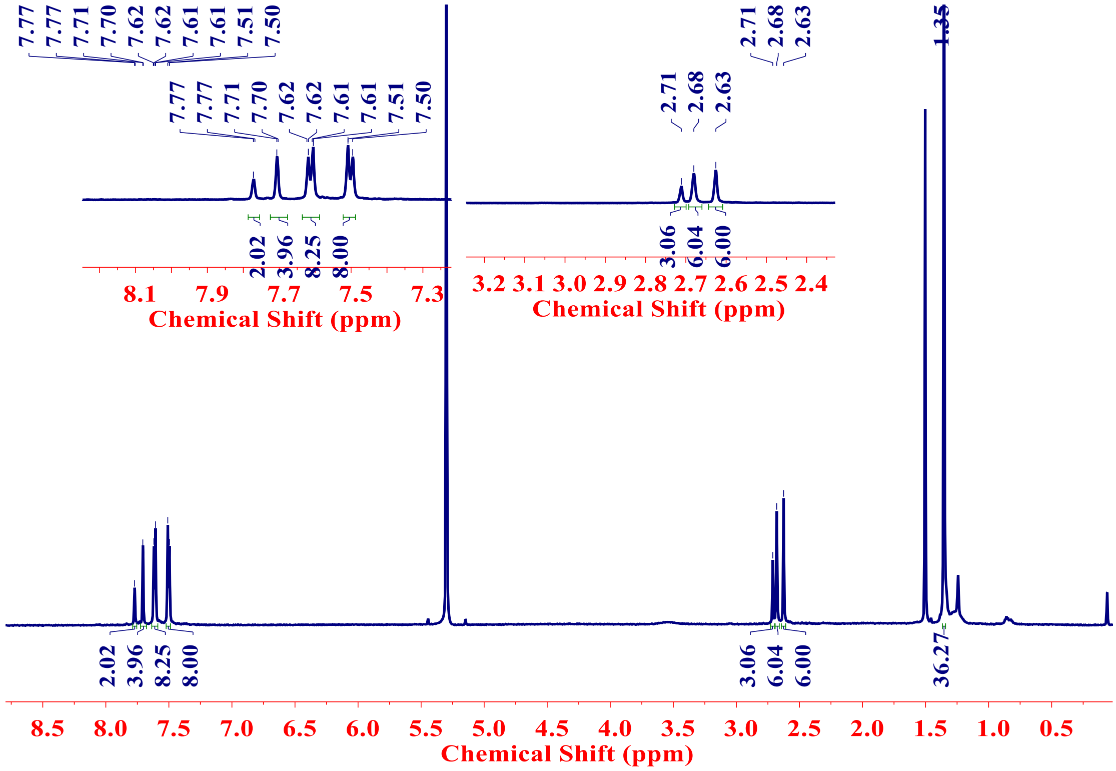


**Figure S46.** $^{1}H$ NMR of compound **9** (400 MHz, $CD_2Cl_2$).

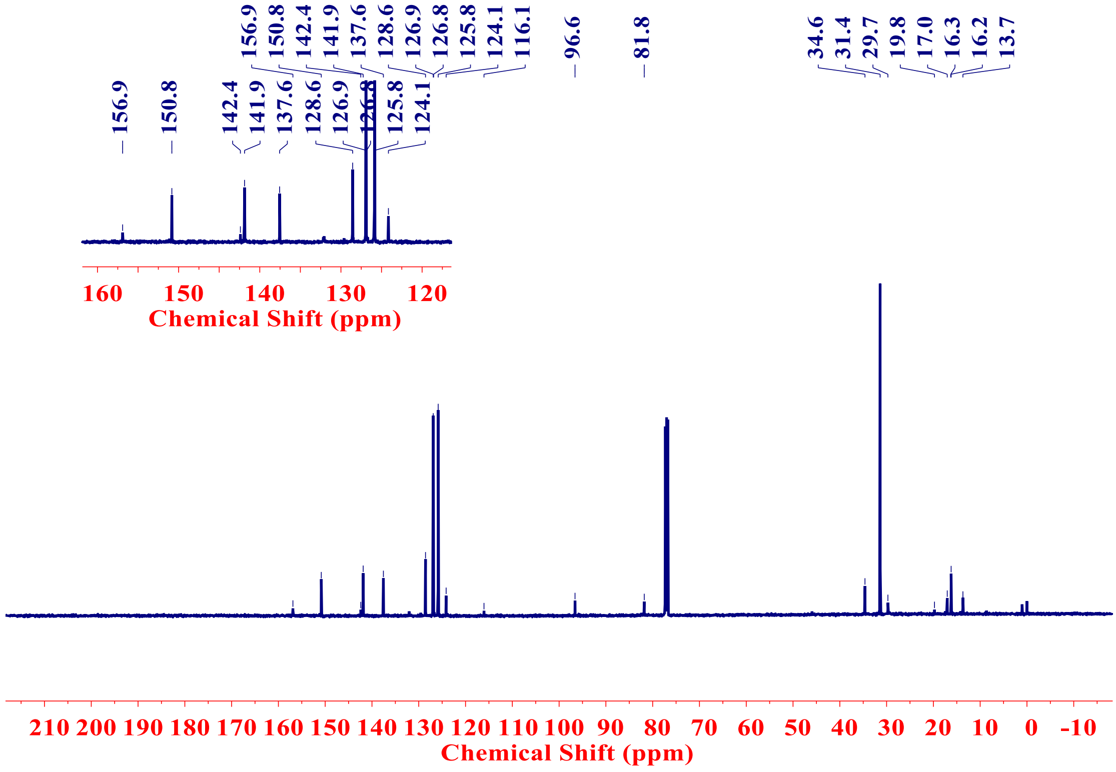


**Figure S47.** $^{13}C$ NMR of compound **9** (125 MHz, $CDCl_3$).

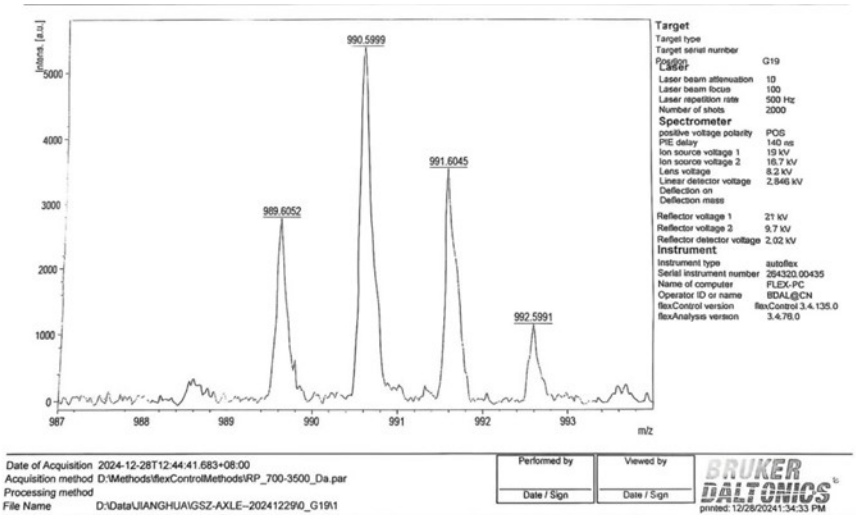


**Figure S48.** MS spectrum of **9**.

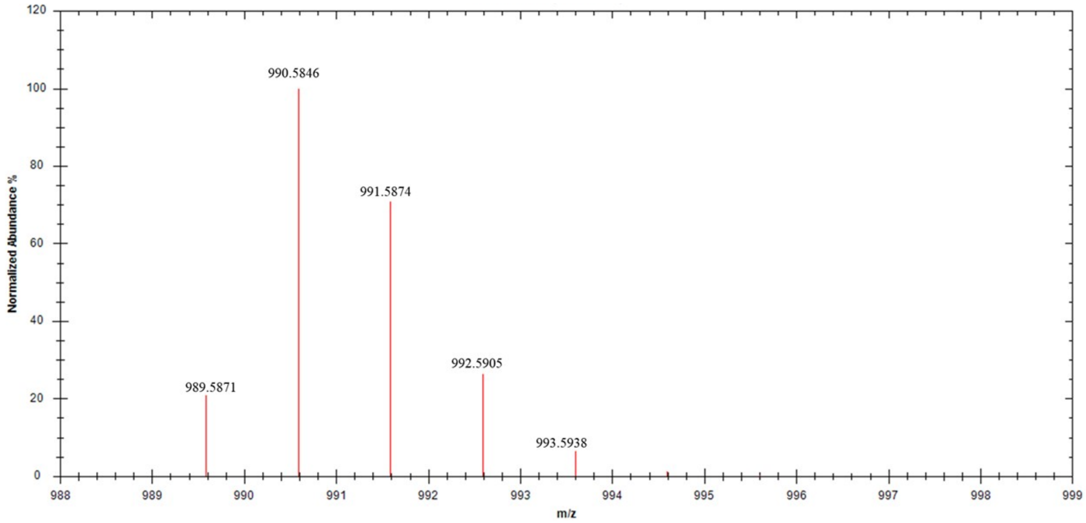


**Figure S49**. Simulated HR-MS spectrum of **9**.

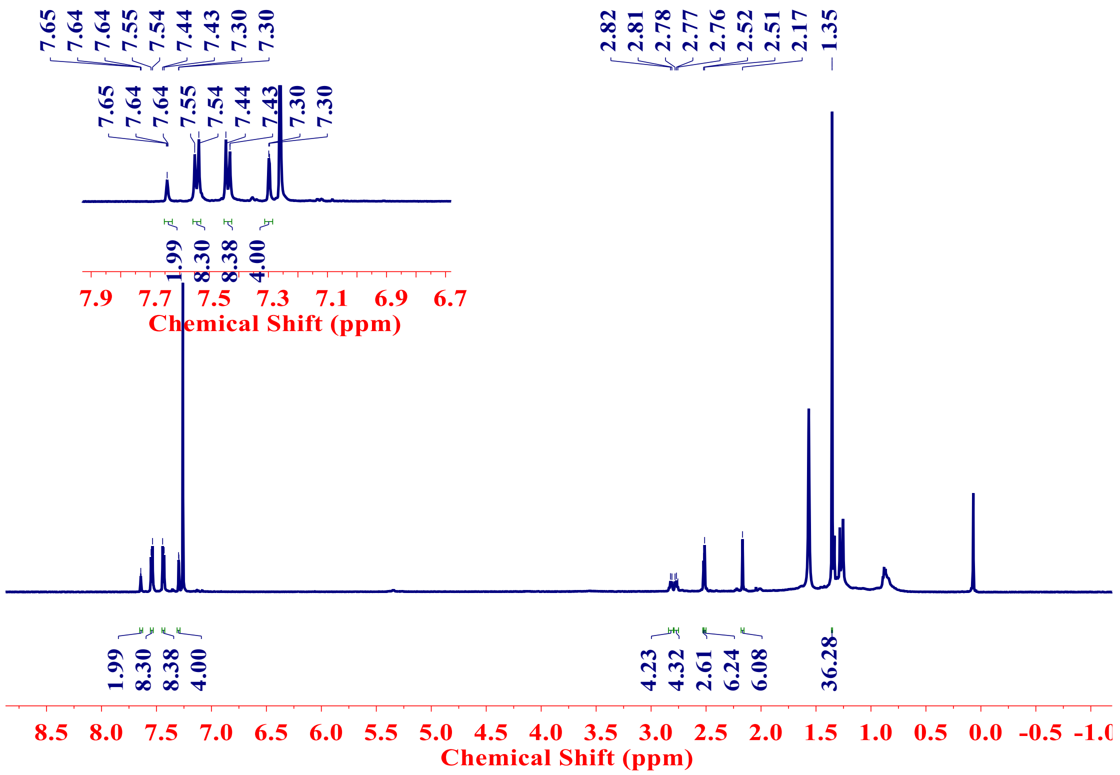

**Figure S50.** $^1$H NMR of compound **10** (400 MHz, $CDCl_3$).

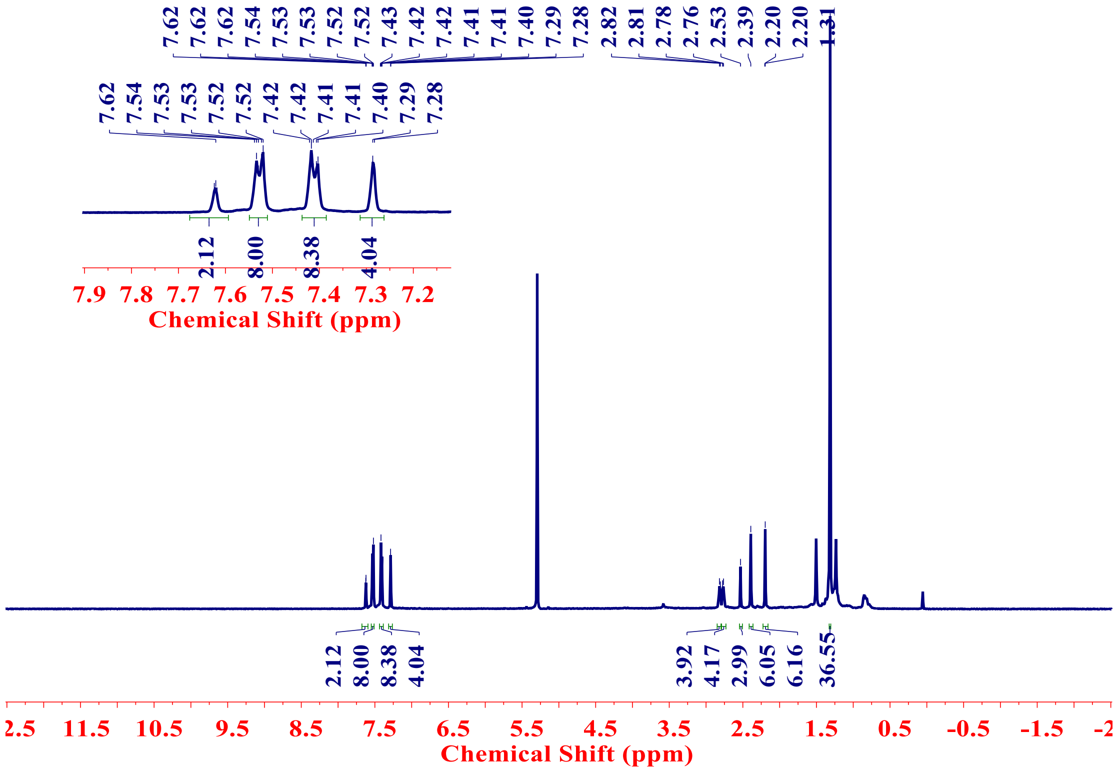

**Figure S51.** $^1$H NMR of compound **10** (400 MHz, $CD_2Cl_2$).

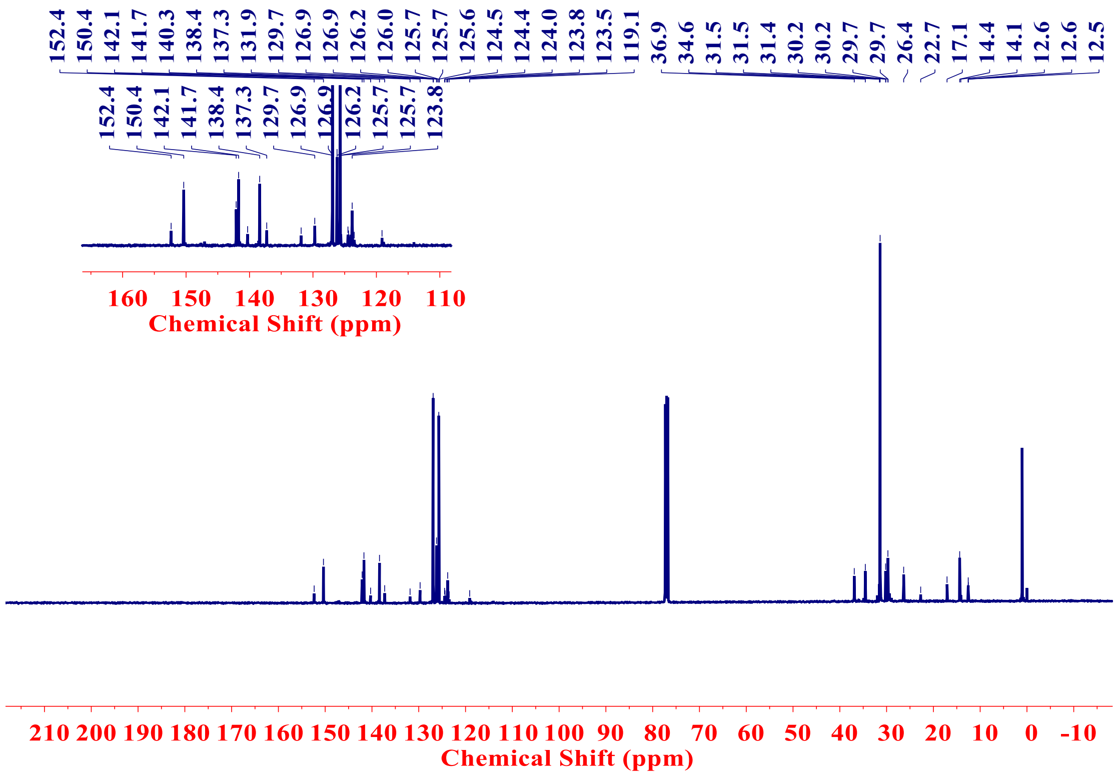


**Figure S52.** $^{13}C$ NMR of compound **10** (125 MHz, $CDCl_3$).

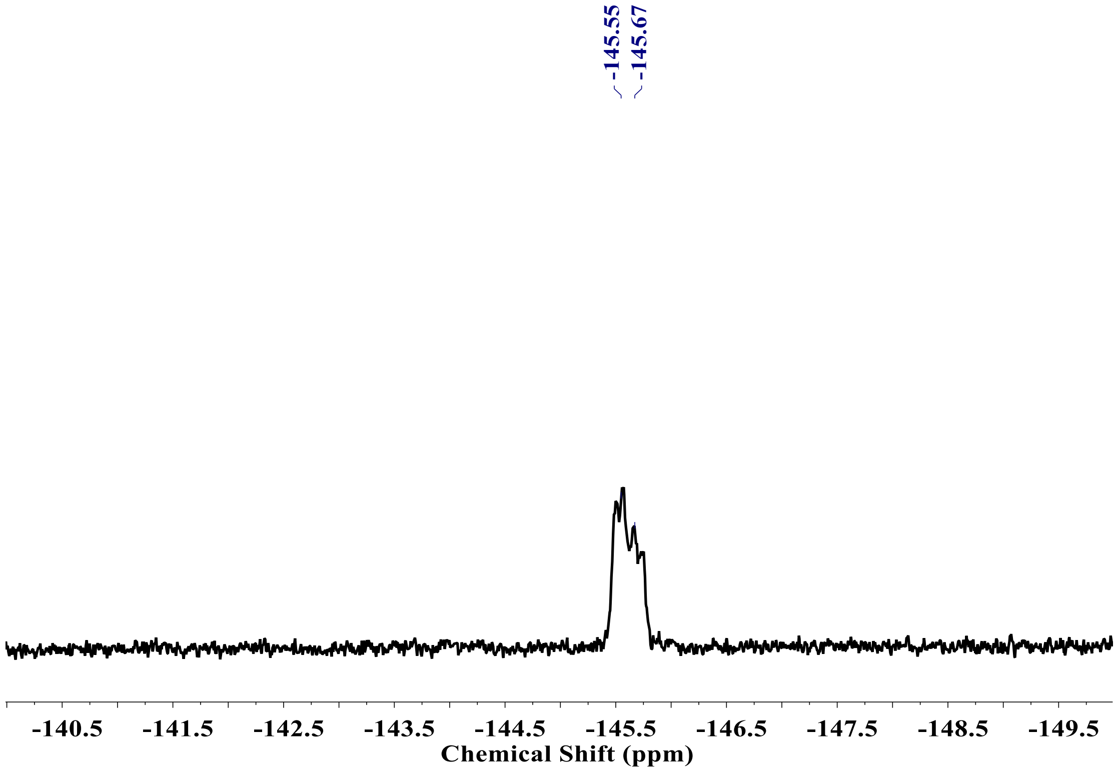


**Figure S53.** $^{19}F$ NMR spectrum of **10** (377 MHz, $CD_2Cl_2$).

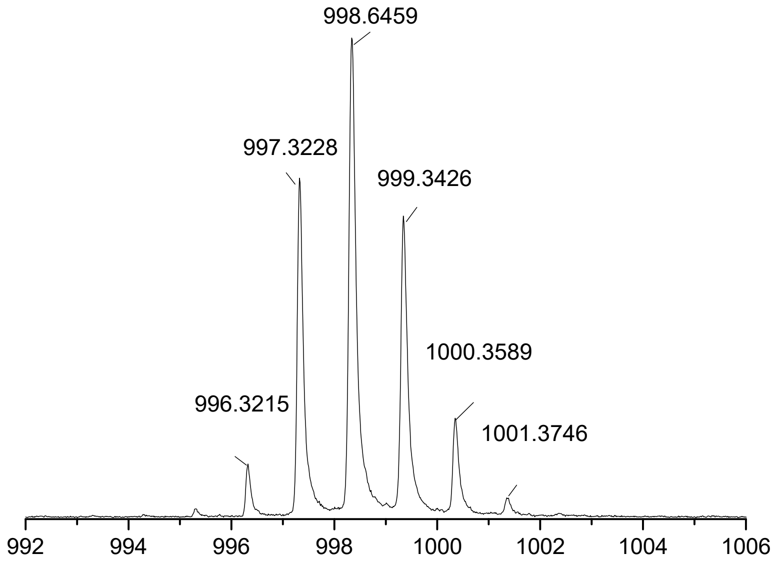


**Figure S54.** MS spectrum of **10**.

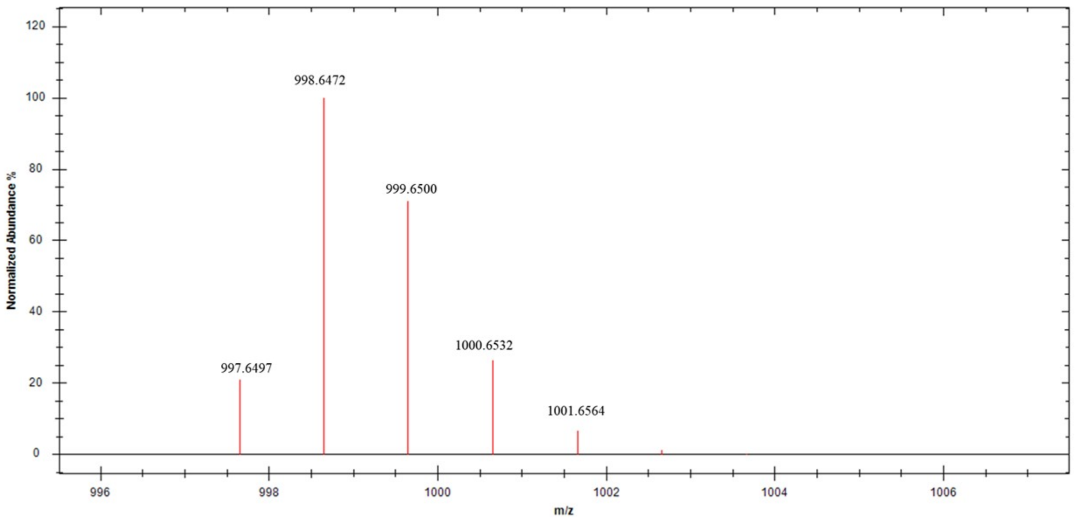


**Figure S55**. Simulated HR-MS spectrum of **10**.
